# A Comprehensive Panel of Three-Dimensional Models for Studies of Prostate Cancer Growth, Invasion and Drug Responses

**DOI:** 10.1371/journal.pone.0010431

**Published:** 2010-05-03

**Authors:** Ville Härmä, Johannes Virtanen, Rami Mäkelä, Antti Happonen, John-Patrick Mpindi, Matias Knuuttila, Pekka Kohonen, Jyrki Lötjönen, Olli Kallioniemi, Matthias Nees

**Affiliations:** 1 Medical Biotechnology Knowledge Centre, VTT Technical Research Centre of Finland, Turku, Finland; 2 Knowledge Intensive Services, VTT Technical Research Centre of Finland, Tampere, Finland; 3 Biotechnology Centre, University of Turku, Turku, Finland; 4 Institute for Molecular Medicine Finland (FIMM), University of Helsinki, Helsinki, Finland; National Cancer Institute, United States of America

## Abstract

Prostate epithelial cells from both normal and cancer tissues, grown in three-dimensional (3D) culture as spheroids, represent promising *in vitro* models for the study of normal and cancer-relevant patterns of epithelial differentiation. We have developed the most comprehensive panel of miniaturized prostate cell culture models in 3D to date (n = 29), including many non-transformed and most currently available classic prostate cancer (PrCa) cell lines. The purpose of this study was to analyze morphogenetic properties of PrCa models in 3D, to compare phenotypes, gene expression and metabolism between 2D and 3D cultures, and to evaluate their relevance for pre-clinical drug discovery, disease modeling and basic research. Primary and non-transformed prostate epithelial cells, but also several PrCa lines, formed well-differentiated round spheroids. These showed strong cell-cell contacts, epithelial polarization, a hollow lumen and were covered by a complete basal lamina (BL). Most PrCa lines, however, formed large, poorly differentiated spheroids, or aggressively invading structures. In PC-3 and PC-3M cells, well-differentiated spheroids formed, which were then spontaneously transformed into highly invasive cells. These cell lines may have previously undergone an epithelial-to-mesenchymal transition (EMT), which is temporarily suppressed in favor of epithelial maturation by signals from the extracellular matrix (ECM). The induction of lipid and steroid metabolism, epigenetic reprogramming, and ECM remodeling represents a general adaptation to 3D culture, regardless of transformation and phenotype. In contrast, PI3-Kinase, AKT, STAT/interferon and integrin signaling pathways were particularly activated in invasive cells. Specific small molecule inhibitors targeted against PI3-Kinase blocked invasive cell growth more effectively in 3D than in 2D monolayer culture, or the growth of normal cells. Our panel of cell models, spanning a wide spectrum of phenotypic plasticity, supports the investigation of different modes of cell migration and tumor morphologies, and will be useful for predictive testing of anti-cancer and anti-metastatic compounds.

## Introduction

Two-dimensional (2D) monolayer cell cultures represent highly reductionist models of epithelial cells and epithelial cancers, due to the loss of physiological extracellular matrix (ECM) on artificial plastic surfaces, and high serum concentrations. Consequently, cells lose relevant properties, such as differentiation, polarization, cell-cell communication and extracellular matrix contacts, while wound healing, inflammatory processes, and hyper-proliferation are artificially promoted. In monolayer culture of prostate cancer (PrCa) lines, the homeostasis of undifferentiated tumor stem cells through basal, transit-amplifying and terminally differentiated, hormone-sensitive luminal cells depends on cell culture conditions, calcium and serum concentration [1], [2], and only poorly represents tumor cell biology in vivo. The lack of a relevant basal lamina (BL), defective ECM deposition, and missing stromal or myoepithelial components [3] further contribute to the artificial nature. As a result, the most effective small molecule inhibitors in monolayer cultures are chemotherapeutic drugs that target proliferation and mitosis. This imbalance contributes to the poor predictive value of compound efficacies between *in vitro* and *in vivo* experiments. Drug action that relates to cell-cell interaction, maturation, epithelial to mesenchymal transition (EMT) and cancer stem cells (CSC) is likely to go undetected. Both 3D architecture and the ECM exert strong effects on drug efficacy [4]. Glandular epithelial cancer cells rapidly adapt to different microenvironments and can dynamically switch between alternative pathways that regulate proliferation, differentiation and survival.

The development of drug resistance or failure to respond to chemotherapeutic drugs also requires appropriate cell culture models. Drug resistance is often attributed to the cancer stem cell (CSC) hypothesis: anti-mitotic cancer drugs spare the slow proliferating, tumor-regenerating stem- or progenitor cells [5]–[7], which eventually re-constitute the tumor mass. This may be concomitant with EMT [7]–[10] and increased metastatic potential [8]. The search for anti-cancer drugs has thus entered a new stage in which researchers increasingly utilize organotypic model systems to more directly explore drug targets on multicellular organoids, often enriched for stem cells [11]. Appropriate *in vitro* experimental models suitable for the analysis of CSC homeostasis, EMT, invasion and metastasis, are becoming increasingly relevant for cancer drug discovery. These should also be cost effective and provide sufficient throughput for high content screening.

The culture of glandular epithelial (cancer) cells in purified ECM, such as collagen, hydrogels or Matrigel, was established over two decades ago [12]. Matrigel represents a reconstituted, laminin-rich basement membrane, which supports processes such as cell polarity, cell-cell- and cell-matrix interaction, and re-expression of differentiation markers even in transformed lines [13]. Mammary and prostate epithelial cells form spheroids, referred to as mammospheres [14] or prostaspheres [15], respectively. Normal prostate epithelial cells differentiate into well-polarized hollow spheroids, a hallmark of functional, glandular epithelial cells. The same microenvironment also supports cell migration, branching and the formation of characteristic acini [16], [17]. In contrast, tumor cells usually show a defective differentiation program, and form atypical spheroids with disorganized architecture, as demonstrated most prominently for breast cancers [18]–[20]. Gene expression patterns of spheroids were demonstrated to correlate with the characteristic phenotypes formed in 3D cultures [19], [20] and overall differentiation and aggressive potential of cancers [21], [22]. Similar to normal epithelial cells, PrCa cells can also actively invade the surrounding matrigel, although their mode of migration is different from the normal, collective sheet or tube migration patterns observed in branching of normal cells. The phenotype of cancer invasion depends on composition and density of the ECM, and can vary from amoeboid blebbing, mesenchymal fibroblast-like motility and multicellular streaming or chain migration [23], [24]. Naturally, the invasive potential also depends on the genetic background of the PrCa cells and their (usually compromised) capability to engage in stringent epithelial cell-cell contacts. Mammary and other epithelial cancer cells form cylindrical, spindle-like cells with the potential to contract and elongate, supporting migration through the surrounding ECM mesh. Much less is known about PrCa. Invasion is assisted by proteolytic processes and proteases such as cathepsins [25], matrix metalloproteinases (MMPs [24]), soluble factors secreted by fibroblasts or the presence of fibroblasts themselves [26], [27], and other factors such as fibronectin and lysyl oxidases [26]. In this regard, 3D models of tumor-cell invasion [28] represent cellular dynamics and architecture of tumors far better than 2D monolayer cultures in which cells spread and glide across the plastic surface. The potential to undergo an EMT and to acquire mesenchymal migration modes is another parameter postulated to contribute to breast-and PrCa invasion and motility [29].

Furthermore, it is unclear if PrCa spheroids, particularly when grown in lrECM, show enrichment of CSC populations (which is often associated with an EMT), or develop resistance against chemotherapeutic agents and ionizing radiation [30], [31]. At the least, involvement of CSC's or EMT would be expected to display a very different dynamics in differentiating 3D cultures in LrECM, compared to floating prostaspheres and 2D monolayer conditions [32]. Last not least, cell culture models for tumor cell invasion are currently restricted to a few widely used, potentially artificial assays (transwell invasion assays; scratch-wound migration assays). Since invasion is fundamentally different under 3D conditions, any representative 3D invasion models represent a veritable novelty [26, 28, and 33].

We report here the development and morphological characterization of miniaturized 3D cell culture model systems, utilizing a panel of 29 prostate cell lines. A selection of the most representative lines were then further characterized by genome-wide transcriptome analyses and systems biology to identify key pathways, signaling molecules, gene networks, and putative drug targets critical for growth and invasion of malignant PrCa cells. Furthermore, bioinformatic image analysis tools to quantify dynamic phenotypic features such as invasive structures, spheroid shape or drug responses have been developed.

## Materials and Methods

### Cell lines and monolayer cultures

Cell lines were purchased from ATCC or requested from the originator laboratories (Table S1). Normal epithelial cells and derivatives (PrEC, EP156T, RWPE-1, RWPE-2, RWPE-2/w99, WPE1-NB14, PWR-1E, PZ-HPV-7 and CA-HPV-10) were cultured in Keratinocyte Serum-Free Medium (KSFM, Gibco), supplemented with 12.5 mg/l bovine pituitary extract and 1.25 µg/l EGF. For 3D cultures, 2% fetal bovine serum (FBS, Gibco) were added. Most PrCa lines were cultured in RPMI-1640 (Sigma-Aldrich), supplemented with 10% FBS. MDA-PCa-2b and NCI-H660 cells were cultured in Ham's F12 medium (Gibco) with 20% FBS, 25 ng/ml choleratoxin, 10 ng/ml EGF, 5 µM phosphoethanolamine, 0.1 ng/ml hydrocortisone, 45 nM selenic acid and 5 µg/ml insuline (all from SIGMA). All cells were propagated at 37°C in standard cell culture conditions (5% CO2, 95% humidity). Identity of cell lines was confirmed by arrayCGH (comparative genomic hybridization) on Agilent 244 k human genome arrays, after 10–15 passages cells were discontinued.

#### Miniaturized 3D cultures

Cells were embedded between two layers of Matrigel on uncoated Angiogenesis μ-slides (Ibidi Gmbh, Germany): bottom wells were filled with 10 µl of Matrigel/culture medium (1∶1; 50%) and polymerized at 37°C for 30 min. Cells were then seeded at 20.000 cells/ml density (∼1000 cells/well). After attachment (1–2 hours at 37°C), cells were covered with a second layer of Matrigel/culture medium (1∶4, 25%), allowed to polymerize overnight at 37°C. Cell culture medium was changed every second day.

#### 3D bulk cultures for RNA extraction

Prostaspheres were cultured in Millicell hanging cell culture inserts with 1.0 µm PET transparent membranes (Millipore) on 6-well plates (Costar). Membranes were pre-coated with Matrigel/medium (1∶1) and incubated at 37°C for 1 h, to prevent attachment to the membrane. Cell suspension was mixed 1∶4 with Matrigel, transferred to the coated well, and polymerized overnight at 37°C. Cells were fed every other day with fresh medium from beneath.

#### Cell fixation, immunofluorescence labeling and imaging

Miniaturized 3D cultures were fixed within microwells, using 4% paraformaldehyde, supplemented with 0.8% Triton X-100 (Sigma-Aldrich), 5 mM EGTA and 1 mM MgCl_2_ for 15–20 minutes at RT. Fixed cultures were washed 3 times with PBS and blocked for 1 h with 20% horse serum. Cultures were incubated overnight at 4°C with primary antibodies (listed in Table S2), washed with PBS, and incubated at room temperature for 4 h with secondary antibodies and Hoechst nuclear stain (1∶5000).

3D structures were stained with Calcein AM live cell dye (Invitrogen). Confocal three-dimensional images were taken by using Zeiss Axiovert 200 M with spinning disc confocal unit Yokogawa CSU22 and a Zeiss Plan-Neofluar 5× objective. Z-stacks were acquired with a step-size of 19 µm. Intensity projections were created by SlideBook 4.2.0.7 and NIH ImageJ (http://rsbweb.nih.gov/ij/), further analyzed with VTT Acca software (sensitivity 10; threshold 5, structures less than 500 pixels in area/size filtered out). Box plots were visualized with R. 20x phase-contrast time lapse images were acquired with Incucyte (Essen instruments), pre-processed with ImageJ and analyzed with VTT Acca (sensitivity 30; threshold 5, structures less than 1000 pixels in area filtered out).

#### RNA extraction and microarrays

3D bulk cultures were washed with ice-cold PBS, membranes excised with a scalpel, and spheroids transferred into 6-well plates. Gels were mixed vigorously with 9 ml of 5 mM EDTA in PBS, transferred into 15 ml Falcon tubes, and incubated on a tabletop rocker for 45 min to detach from the Matrigel. Prostaspheres were sedimented by centrifugation and lysed with RLT buffer (Qiagen). Cells propagated in monolayer were lysed at 90% confluence, directly from 10 cm cell culture dishes using RLT buffer. Total RNA (from biological replicates) was extracted with RNeasy Mini kit (Qiagen), according to the manufacturer's protocol. 300 ng RNA was amplified with Ambion's Illumina TotalPrep RNA Amplification kit. IVT reaction was performed overnight (∼16 h) to yield sufficient biotinylated cRNA. RNA and cRNA concentrations were measured with a nanodrop ND-1000, overall quality was monitored with BioRad's Experion electrophoresis station. 750 ng cRNA were hybridized on Illumina's Sentrix HumanRef-8 v3 BeadChips, at 58°C overnight. Hybridized cRNA was detected with 1 mg/ml Cyanine3-streptavidine (GE Healthcare Biosciences), and arrays scanned with Illumina BeadArray Reader. Data were quality checked and extracted using Illumina GenomeStudio software, without normalization or background subtraction.

#### Microarray data analysis

Raw microarray data were quantile-normalized, using the bioconductor R package “beadarray”. Normalized data were further processed using a variance and intensity filter. Statistical analysis of differential gene expression was performed using the limma and lumi R/Bioconductor packages. The threshold for differential expression was q<0.05 after a Benjamini-Hochberg multiple testing correction. Normalized Illumina gene expression data of the entire panel of experiments have been submitted to GEO (Gene expression Omnibus) as study **GSE19426**.

Data were then used in two different modes: to evaluate relative changes of gene expression between 2D and 3D experiments, or different time points in 3D culture, mean normalized values in 3D were subtracted from mean values of replicates in 2D monolayer culture and ratios calculated. Log(2) transformed 2D/3D ratios were then utilized for clustering and heatmap generation (Cluster 2.11 and TreeView 1.60, Stanford University), and gene ontology analysis (GO; DAVID, http://david.abcc.ncifcrf.gov/). K-Means clustering was used to draw representative heatmaps based on 2D/3D ratio data, generating 12 nodes (100 iterations). The Gene Set Analysis (GSA) package in R was used to define significantly enriched gene categories, here the “Maxmean” statistics was used to calculate enrichment scores, and permutation based p-values were derived from 1000 bootstrap replicates. A false discovery rate (FDR) correction was also applied as a measure of relevance. Gene sets utilized for analysis were obtained from the Molecular Signatures Database (MSigDB, Broad Institute), including positional, curated, co-expression neighbourhood, GO, and evolutionarily conserved transcription-factor targets.

Secondly, normalized but otherwise un-processed gene expression data were utilized to define gene signatures that correlate with phenotypic characteristics (round/normal, mass, stellate phenotype). Principal component analysis (PCA) and plotting of informative genes correlating with spheroid morphologies were performed based on specialized R scripts. Genes representing the largest percentage of variance were selected based on ANOVA.

#### Ingenuity Pathway Analysis (IPA) and compound selection

Differentially expressed gene clusters (2D/3D ratios) were uploaded to IPA to perform gene network analyses and identification of potentially informative central “hub” genes. Specific small molecule inhibitors against certain hubs or hub genes and pathways were acquired from TOCRIS and SIGMA-Aldrich (see below). Additional and independent sources of drug/target information were also utilized for the same purpose (DrugBank; http://www.drugbank.ca; Matador http://matador.embl.de).

#### RT-PCR validation

2 µg of total RNA were reverse transcribed with Invitrogen Superscript II reverse transcriptase in 50 µl. cDNAs were diluted 1/10. QRT-PCR was performed in triplicates with the 7900HT Fast Sequence Detection System (Applied Biosystems) in 96-well or 384-well plate format, 8 µl/well. PCR primers and probes were designed based on the Roche Universal Probe Library, oligonucleotides were ordered from Sigma-Aldrich. Primers were used at 300 nM, probes at 100 nM concentration; with 2x TaqMan® Universal PCR Master Mix. PCR runs were analyzed using Applied Biosystems SDS software.

#### Protein lysate microarrays (LMA)

Prostaspheres were collected on days 3, 6, 8, 10 and 14; according to the following protocol: microwells were washed with PBS, Matrigel mixed with ice-cold 5 mM EDTA in PBS, transferred into v-bottom 96-well plate (Greiner), and incubated on ice in a tabletop-shaker for 30 minutes. Spheres were sedimented by centrifugation and lysed in LMA-buffer (0.2% SDS, 100 mM Tris, 10 mM DTT). Monolayer cells were harvested in LMA-buffer at 90% confluence in 10 cm plates. For each time point, two biological replicates were printed on a single array. Printing, staining, scanning, background subtraction, normalization relative to β-actin signal, and data analyses were performed as described previously [34].

#### Western blotting

Protein samples from culture wells were collected as described from microwell plates, and lysed in WB-buffer (25 mM Hepes pH 7.5, 100 mM NaCl, 5 mM EDTA pH 8.3, 0.5% Triton X-100, 20 mM β-glycerophosphate, 100 µM orthovanadate, 0.5 mM PMSF and 1 mM DTT). Protein concentration was measured by Bradford assay, and proteins separated by SDS-PAGE with precast PAGEr gels (Lonza), transferred on Protran nitrocellulose transfer membrane (Whatman), and blotted with the primary antibodies listed in Table S3. Multiplex incubation with three antibodies was used to accommodate for the small total amount of proteins extracted from miniaturized cultures. Antibodies were detected with Alexa infrared dye-conjugated secondary antibodies (Invitrogen), and membranes scanned with the Odyssey Infrared Imaging System (LI-COR).

#### Drug treatments in 3D

compounds were ordered from SIGMA or Tocris Inc., and dissolved in the appropriate vehicle (DMSO, Ethanol, 1xPBS) according to manufacturer's instructions. Recombinant human chemokines, cytokines, and function-blocking antibodies were ordered from R&D Systems. Drugs were prepared as 10 mM stock solutions, stored at −20. Most chemokines and peptides were diluted to 1 µg/µl stock solutions. Dilution to working solutions was done immediately prior to treatment. Drugs were added after a 4 day period, during which spheroids develop, and maintained for up to 7 days. Drug concentrations were selected according to half maximal inhibitory concentration (IC50), known for most compounds. All treatments were performed in triplicates. Spheroids were monitored in real-time by live-cell imaging (Incucyte, Essen Instruments; 10x objective), acquiring 1 image/h.

#### Cell proliferation assays

Cells were seeded on 384-well plates 24 h before the drugs were added. After 72 h the number of living cells was assessed with CellTiter Blue® Cell Viability Assay (Promega) according to manufacturer's protocol. Fluorescent signal was quantified with EnVision Multilabel Plate Reader (Perkin Elmer).

## Results

### Normal prostate epithelial cells and PrCa lines form characteristic morphologies in Matrigel

**Figure 1:**
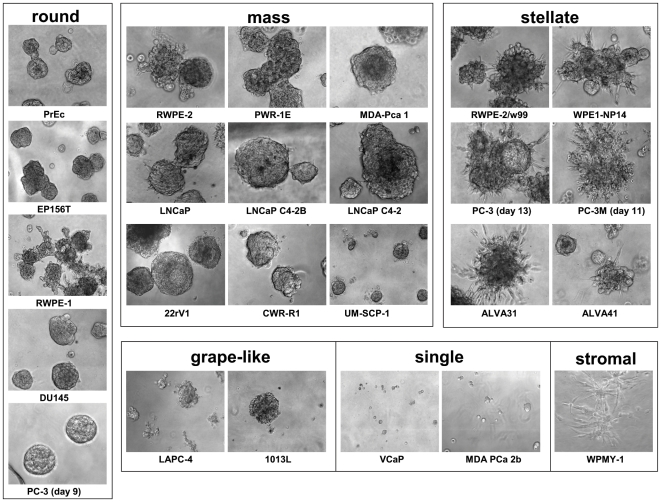
Representative phase contrast images of spheroids formed in 3D Matrigel culture. Most structures fell into the categories round, mass, stellate, or grape-like. Some cell lines failed to form spheroids in Matrigel but persisted as single live cells for up to two weeks (single phenotype). Most spheroids were imaged at days 9–10 after inoculation. PC-3 spheroids, shown as round phenotype at day 9, undergo a metamorphosis to invasive/stellate phenotype at day 13. The same occurs to PC-3M at an earlier time point (day 5–8).

Normal prostate and prostate cancer (PrCa) cell lines fail to differentiate and form multicellular structures in purely collagen-rich extracellular matrix (Figure S1). In collagen, both normal- and tumor cells formed only loose aggregates, with poor or no cell-cell contacts, often displaying a fibroblast-like growth pattern. In contrast, Matrigel strongly supports both growth and differentiation of normal and PrCa spheroids. Matrigel has profound effects on all cell lines tested and, with few exceptions; formation of relevant multicellular structures is supported. Spheroid formation in Matrigel was typically initiated by single cells. The spheroids formed in Matrigel generally fell into four morphological categories, adapted from [19], [35] (Figure 1; Figure S2).

#### Branching/Round phenotype

**Figure 2:**
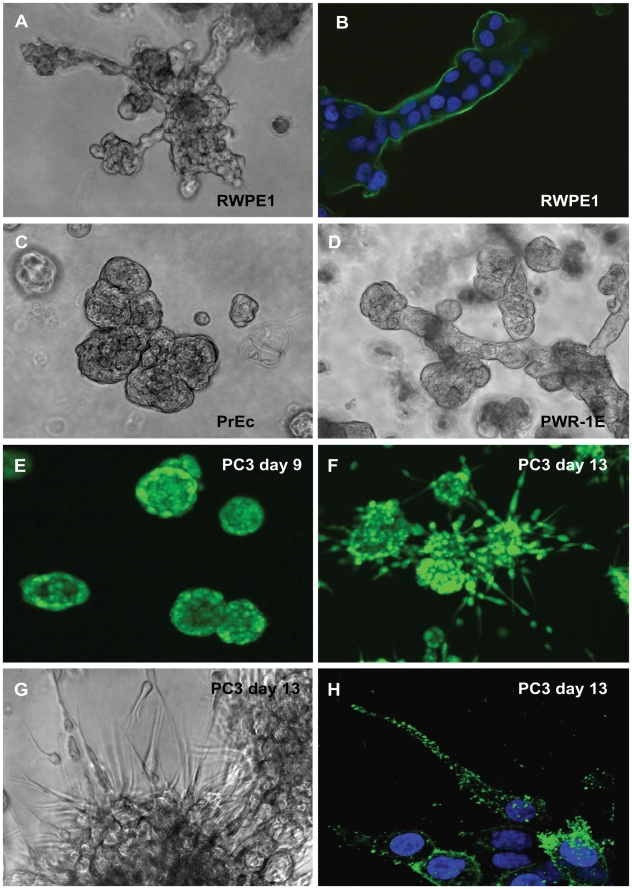
Phase contrast and confocal laser scanning microscopy of various prostate cells and cell lines grown in Matrigel, illustrating the phenotypic differences between branching phenotype typically observed in several non-transformed cell lines, and the stellate/invasive phenotype restricted to tumor cells. A) Phase contrast image of branching structures formed by RWPE-1 cells, B) acinar structure formed by RWPE-1 stained with an antibody against laminin B1. C) Branching or cluster-like structures formed by primary prostate epithelial cells (PrEC). D). Mixed branching and mass phenotype structures, formed by PWR-1E cells. E) Round structures formed by PC-3 cells at day 9, live cell staining with calcein. F) Morphological transformation of round spheroids into invasive structures at day 13. G) Phase-contrast image of invasive PC-3 structures around day 13. H). Staining of PC-3 filopodia with an antibody specific for the active form of integrin beta 1 (ITGB1), illustrating dense decoration of spindle-like cell structures.

**Figure 3:**
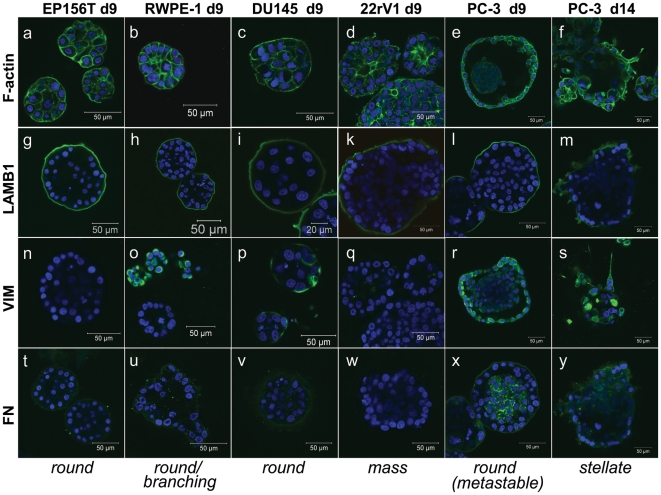
Representative immune cytochemistry staining of spheroids structures, grown for 9 or 14 days in Matrigel, using antibodies against the basal-lamina component laminin beta 1 (LAMB1), and the mesenchymal markers vimentin (VIM), and fibronectin (FN). Alexa488-labeled phalloidin was used to detect F-actin and Hoechst stain to label nuclei (blue). RWPE1, DU145 and PC3 cells show expression of EMT-related mesenchymal markers, regardless of phenotype (PC-3 cells; round at day 9, stellate at day 14).

Normal primary prostate epithelial (PrECs) and non-transformed lines such as RWPE-1 and EP156T cells formed round spheroids after 6–10 days in culture (Figure 1). Normal PrECs and in-vitro immortalized cell lines such as RWPE-1 and PWR-1E cells simultaneously formed branching acinar and round spheroid structures, actively migrate into the surrounding ECM in the form of large cell aggregates (Figure 2a–d). EP156T cells showed no or few branching structures. Round structures generally developed a robust basal lamina (BL), encapsulating both spheroids and acinar structures (Figure 2, Figure S2, Figure 3g–m). Surprisingly, the tumor lines DU145, PC-3 and PC-3M cells also formed round and well differentiated, polarized spheroids (Figure 2e), surrounded by a complete BL, and frequently containing a lumen (PC-3; Figure 3e+r). Additionally, PC-3 spheroids often contained an internal cell mass reminiscent of structures seen in PIN (prostate intraepithelial neoplasias). Immune staining for tight-junction proteins such as ZO-1 (not shown) and F-actin (Figure 3a–c) demonstrated generally very robust cell-cell contacts and cell polarization in round spheroids formed by both normal and tumor cells.

#### Mass phenotype

the majority of PrCa (LNCaP, 22rV1, MDA-PrCa 1, UM-SCP1, CWR-r1, LAPC-4) and two *in vitro* transformed lines (PWR-1E, RWPE-2) generated large, irregular spheroids with often incomplete or missing BL, also lacking a hollow lumen (Figure 3d+k). PWR-1E was the only mass-phenotype cell line capable of branching/acinar morphogenesis (Figure 2d). The luminal keratins KRT8 and KRT18 were always strongly expressed (Table S3). Cell-cell contacts, maturation and polarization were generally less pronounced, compared to round spheroids, reflected in the often kidney shaped irregular spheroids (Figure 1). Mass phenotype structures did usually not show invasion of the lrECM; however, formation of filopodia or pseudopodia was consistently observed in the 22rV1 and occasionally in the LNCaP and RWPE-2 cell lines. In LNCaP spheroids, cells were frequently observed to leave the spheroid structures at sites of incomplete BL coverage (Figure S2).

#### Grape-like phenotype

Only one cell line, 1013L, consistently formed loose clusters of cells with particularly poor cell-cell contacts, lacking any BL. LAPC-4 cells formed both mass and grape-like structures. No invasive properties were observed in these cell lines.

#### Stellate “invasive” phenotype

The in vitro transformed cell lines RWPE-2/w99, WPE-1/NB14, and the tumor lines ALVA-31 and ALVA-41 formed stellate or invasive structures, characterized by spindle-like filopodia (Figure 2g) and the rapid migration of chains of cells through the surrounding ECM. Invasive structures formed were almost exclusively multicellular and showed a chain-like invasion mode. Fibroblast-like, mesenchymal invasion of single cells was observed only occasionally. The *in vitro* transformed lines RWPE-2, RWPE-2/w99 and WPE1/NB14 simultaneously formed stellate structures and round spheroids, indicating heterogeneous composition of these cell lines. Of these, RWPE-2/w99 represented the cell line with the most consistent stellate phenotype, and was selected for further experiments. Immortalized prostate stromal cells (WPMY-1) and tumor-derived, primary stromal cells (not shown) also formed stellate-like structures, however lacking rapid motility and invasive properties.

#### Invasive switch

Round and well differentiated, polarized spheroids were formed by PC-3 and PC-3M cells, but underwent a spontaneous transformation towards invasive morphology around 10–13 and 6–8 days in 3D, respectively (Figure 2e+f, supplemental invasion Movie S1). The onset of morphological transformation into the stellate, invasive phenotype was dependent on cell density. Transformation could be temporarily delayed and even partially reverted upon feeding fresh medium, but eventually continued to progress until all structures were thoroughly transformed and only stellate structures remained (Figure 2g). Invasive structures and filopodia formed even prior to invasion strongly expressed the active form of the laminins-receptor integrin beta 1, indicating strong contacts to the extracellular matrix as a prerequisite for invasive processes (Figure 2h). Simultaneously, the BL of transformed structures becomes increasingly fuzzy and disintegrated (Figure 3m). Strong expression of mesenchymal markers Vimentin VIM and Fibronectin FN1 (Figure 3o–y), observed in non-invasive RWPE-1 (heterogeneous) and DU145, but also in PC-3 cells, did not correlate with the stellate phenotype. Furthermore, expression of VIM and FN1 were not increased after the invasive transformation of PC-3 and PC-3M cells (Figure 3s+y)

#### “Single” phenotype

Some cancer lines (VCaP, DuCaP, NCI-H660 and MDA-PCa 2b) failed to form spheroids, but persisted as single cells (“single” or “singular phenotype”) for up to 2 weeks. Interestingly, all of these cell lines were positive for ETS-transcription factor fusion events or rearrangements (TMPRSS2-ERG in H660, VCaP and DuCaP, a balanced ETV1 rearrangement in MDA-PCa 2b). Gene expression analyses of VCaP cells in Matrigel indicated that the cells may undergo terminal differentiation or senescence when embedded in Matrigel (data not shown). Expression of the TMPRSS2-ERG fusion gene and proliferation-relevant genes was reduced in Matrigel. However, growth of VCaP and DuCaP was not restricted in collagen type I gels; and gene expression patterns in Col I were limited (not shown).

### Dynamic changes of gene expression in response to Matrigel correlate with normal, transformed and invasive properties

**Figure 4:**
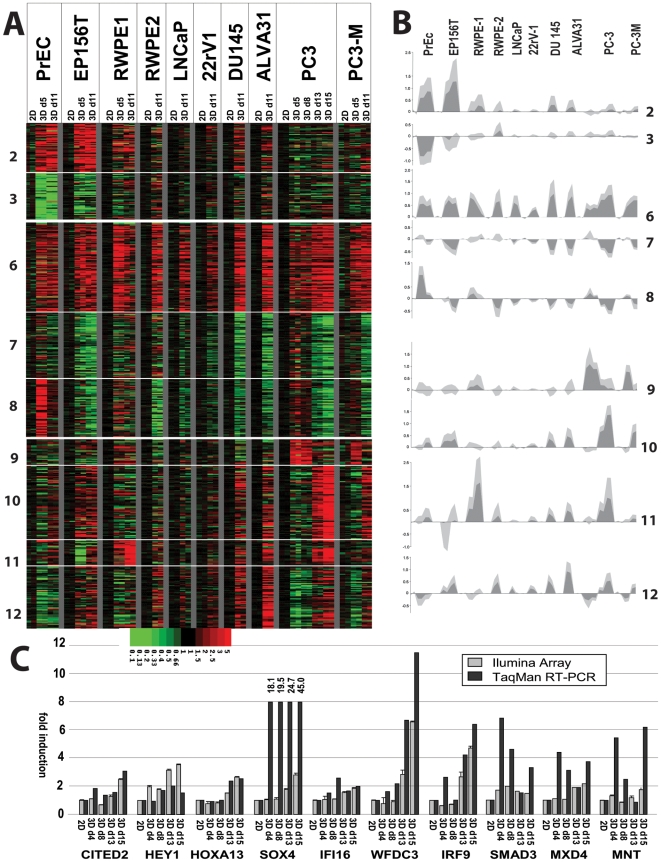
Characteristic patterns of altered mRNA gene expression of cells cultured in laminin-rich ECM (Matrigel), compared to monolayer culture (ratios 3D/2D). A) Heatmap, illustrating 12 clusters generated by the K-means algorithm. Clusters 1+2 represent genes exclusively affected in normal cells (PrEC, EP156T). Clusters 6–8 represent genes similarly induced or repressed in all cell lines in response to 3D cell culture in Matrigel 3D, regardless of phenotype, or transformation. Clusters 9–12 contain genes characteristic for the stellate/invasive type cell lines (ALVA31, PC3, PC3M, RWPE2/w99), or which are induced during the spontaneous conversion of round to stellate structures (e.g. PC3, day 13 and 15). B) Average expression changes, illustrating the median (dark grey) and variation (light grey) of altered gene expression within clusters. C) QRT-PCR analysis (black) and Illumina array data (grey) for a selected panel of genes, validating increased expression in invasive PC-3 cells.

LrECM and the formation of spheroids induce fundamental changes in cell biology, protein and mRNA gene expression of PrCa cells. About 3400 mRNAs were differentially expressed between 2D and 3D conditions, however not consistently across all cell lines and all time points. Three generalized patterns of altered gene expression were observed across the panel of cell lines (Figure 4a+b). Altered expression of selected genes was validated by qRT-PCR (Figure 4c). Factors of differential expression, as confirmed by qRT-PCR, were generally greater compared to the array data. GO analyses and GSEA revealed highly significant enriched functional gene categories for most of the clusters (Tables S4, S5 and S6).

#### a) Non-transformed cells

Genes whose response to 3D Matrigel culture (cluster 1+2; Figure 4a; Table S4) was restricted to non-transformed cells were mainly related to ECM turnover, lipid and eicosanoid/prostaglandin metabolism, or cell differentiation. These gene sets are likely to be required for both normal spheroid maturation and acinar branching, and include known regulators of epithelial differentiation, cell migration and acinar morphogenesis such as WNT5A [36], [37] and the basal-type cytokeratins suchas KRT5 and KRT14. A number of these genes were associated with basal epithelial differentiation patterns. In contrast, PrCa cells preferentially show luminal differentiation.

#### b) Generalized Effects of Matrigel on Gene Expression

Gene sets that homogeneously respond to lrECM, regardless of the cell line, transformation status or spheroid morphology fell into 3 clusters: Cluster 7 (repressed) was highly enriched in mitochondrial and ribosomal functions, mRNA processing, and general metabolic processes, indicating the overall reduced growth, metabolic activity and proliferation of cells in 3D compared to monolayer culture. Similarly, cluster 8 showed an extremely significant (p<10^−57^) enrichment of cell-cycle, DNA-synthesis, mitosis, and proliferation processes, confirming the general reduction of cell proliferation in response to lrECM. However, the average fold change (Figure 4b) observed for these genes ranged between 1.5 to 2 fold; indicating that cells in 3D culture continue to replicate, however more slowly compared to 2D. Normal PrEC's continue to proliferate in lrECM somewhat longer compared to PrCa lines; this effect has also been described for primary mammary epithelial cells [18]. Cluster 6 was highly enriched in genes related to lipid/steroid metabolism (p<10^−11^), chromatin modification and epigenetic re-programming (p<10^−7^), pointing to profound epigenetic changes involved in acinar differentiation.

#### c) Invasive transformation

Gene sets expressed in stellate or induced during the morphological transformation of round PC-3 spheroids into stellate structures (cluster 9) were enriched in GO terms related to cell adhesion, cell-cell contact, invasion/metastasis, and ECM turnover (collagens, laminin isoforms, integrins ITGB2, ITGB4, ITGAV, ITGA1 and ITGA10; Table S4). This cluster also contained many early developmental transcriptional regulators (CITED2, HEY1, SOX4, SOX9, FOXO3, HOXA13, HOXB13, qRT-PCR validation in Figure 4c). Cluster 11, showing strong induction of genes in both invasive PC-3 and branching RWPE-1 cells, contained mainly interferon-inducible genes (OAS1, OAS2, MX1, IFI27, STAT1, STAT2 etc). This may suggest a dual role of IFNs α/β, STAT1/STAT2 transcription factors and inflammatory processes in both invasion and branching of non-transformed epithelial cells (RWPE-1).

### Principal Component Analysis: mRNA gene expression signatures of cell lines correlate with the Morphology in 3D

**Figure 5:**
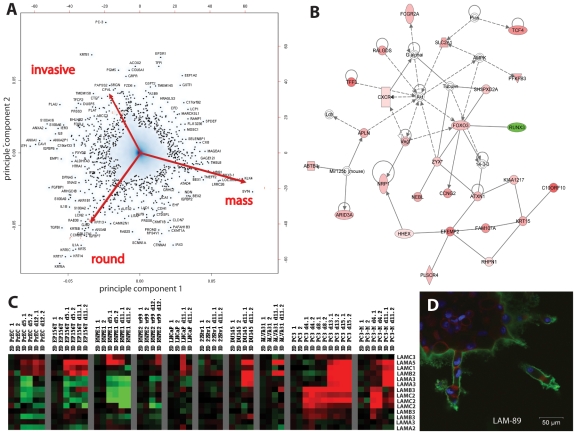
Functional analysis of altered gene expression data in 3D culture, compared to 2D monolayer. A) Principal component analysis (PCA), illustrating gene sets that distinguish round, mass-, and stellate morphologies. Round phenotype cells included were PrEC, EP156T, and RWPE1. The mass phenotype is represented by LNCaP and 22rV1 cells. Stellate/invasive cells comprise ALVA31, PC3, PC3M, and RWPE-2/w99. B) Ingenuity Pathway Analysis (IPA) of invasion-related clusters 9–12 (Figure 4), illustrating network 2 out of 13 generated. This network is centered on the AKT and PI3-Kinase pathways. Colors correlate to factors of differential expression observed between round and invasive spheroids (red: induced, green: repressed). C) Heatmap, illustrating altered mRNA expression of laminins in 2D and 3D culture. Expression of laminins α5, γ1, β2 and α3 is strongly induced in the invasive transformation of PC-3 and PC-3M cells (days 13+15). D) Continuous expression of various laminins after onset of invasion, as demonstrated by staining with a human-specific antibody (LAM-89) that detects various alpha and beta subunits.

Principal component analysis (PCA, Figure 5a) was used to identify the most characteristic gene signatures that may distinguish spheroids of normal/round (PrEC, EP156T, and RWPE1), mass (LNCaP and 22rV1) and stellate morphologies (ALVA31, PC3, PC3M, RWPE-2/w99). The basal keratins KRT5, KRT6A-C, KRT13, KRT14, and KRT17 represent the most representative genes for round spheroids, characteristic for the basal-like phenotype of in-vitro immortalized lines and normal prostate-epithelial cells. Luminal markers such as keratins KRT8 and KRT18 were only poorly expressed (Table S3), but inflammatory chemokines such as interleukin 1a and IL1b were also characteristic. In contrast, luminal differentiation-related and androgen-inducible genes such as NKX3-1, SYT4, KLK4, CK18, and TMSL8 were identified as the most characteristic markers for the mass phenotype, which represents the majority of PrCa cell lines. Genes such as CTGF (connective tissue growth factor) or PLAT (tissue-type plasminogen activator) were most characteristic for invasive cell lines like PC-3 or RWPE-2/w99; indicating a possible role of TGF beta signaling, active remodeling of the ECM, and mesenchymal properties during invasion [38]. Further analysis of the genes most strongly associated with invasive/stellate phenotype, using Ingenuity Pathway Analysis (IPA), resulted in multiple gene networks, including one that illustrates an association with the AKT pathway and signaling through various G-protein coupled receptors (G alpha_i_ proteins), chemokines-receptor CXCR4, the invasion- and angiogenesis related Neuropilin (NRP1) and the neuropeptide apelin (APLN). Other connected genes were the cytoskeletal proteins zyxin (ZYX) and nebulette (NEBL), ECM-related genes EFEMP2 (EGF-containing fibulin-like extracellular matrix protein 2), rhophilin (RHPN) and FAM107A, and the transcription factors FOXO3 and TCF4 (Figure 5b). Although the basal lamina of invasive, stellate structures becomes increasingly fuzzy and disintegrated, invasive PC-3, PC-3M and ALVA31 cells continued to secrete a different panel of laminins. While laminin-5 (α3β3γ2), associated with normal epithelial differentiation, was re-induced at early time points in PC-3 cells growing in 3D culture, other laminins subunits (α5, β2, γ1) were de-novo expressed after transformation (Fig 5c), as validated by immune fluorescence (Figure 5d, LAM-89 pan-laminin antibody).

### A role for Epithelial-to-Mesenchymal Transition (EMT) in invasion and the stellate phenotype?

The cell lines with the most prominent latent, invasive potential (ALVA31, PC-3 and PC-3M), to some degree shared by the heterogeneous RWPE-1 and RWPE-2/w99 cells, showed the highest expression of mesenchymal markers (Vimentin VIM, Fibronectin FN1, N-cadherin CDH2 (Figure S3a), CDH11 (Figure S3b), and loss of expression of epithelial markers such as E-cadherin CDH1 (Figure S3c). Simultaneously, mesenchymal and epithelial cadherins were co-expressed in RWPE-1 cells (CDH1 and CDH2; Figure S3a). This indicates that these cells (or subpopulations e.g. within RWPE-1; Figure S3c) may have undergone an epithelial-mesenchymal transition (EMT), possibly in vitro. This observation is further supported by the homozygous deletion of catenin alpha-1 (CTNN1A) in PC-3 and PC-3M, a gene that cooperates with E-cadherin in formation of epithelial cell-cell-contacts (not shown). The loss of PTEN (phosphatase and tensin homolog) in PC-3, PC-3M and ALVA31 cells may have also contributed to this EMT [39], [40] and the concomitant activation of AKT and PI3 Kinase pathways. However, many mesenchymal marker genes (VIM, FN1) and EMT-related transcription factors (e.g. Slug, SNAI2) were strongly expressed in both 2D and 3D culture, remained unchanged throughout all stages of spheroid formation, and were not significantly induced in the invasive transformation of PC-3 spheroids (Figure 3s). Additionally, VIM and FN1 were also expressed in non-transformed RWPE-1 and non-invasive DU145 cells (Figure 3o–y). Slug (SNAI2) shows the highest expression in non-invasive cell lines and may be required for normal prostate differentiation (Figure 5a; Figure S3b). TWIST1 expression correlates more consistently with the EMT-related observations.

High level EMT marker expression may indicate a latent or metastable EMT phenotype, which is temporarily repressed by the lrECM in favor of normal epithelial differentiation. Eventually, mesenchymal phenotypic features prevail, overriding epithelial differentiation patterns which may then result in cell invasion. In contrast to the EMT/mesenchymal markers, many genes downstream of AKT and related cancer-relevant pathways (PI3-Kinase, PTEN, IGF1R, mTOR and S6 Kinase) are induced when PC-3 and PC-3M cells become invasive (Figure S4a–c). Among others, these prominently include the invasion-related integrins alpha 10, beta 4, and beta 2; many laminins and collagen subunits and the interleukins IL10 and IL23A. Clinical gene expression data (Figure S4d) [41] validated that invasion- and AKT/PI3-Kinase associated genes, as exemplified by collagen 1 alpha 1, may also be up-regulated in PrCa compared to normal prostate, and may correlate with high Gleason grade tumors.

### Pathways, key regulatory proteins and molecular mechanisms correlate with spheroid formation and invasion

Key pathways for the formation of round and mass spheroids, in comparison to 2D/monolayer culture, were identified by a combination of multiple bioinformatic approaches, including Principal Component Analysis (PCA), Ingenuity Pathway Analysis (IPA), Gene Ontology annotation (GO), and Gene Set Enrichment Analyses (GSEA).

#### Round and mass phenotype

**Figure 6:**
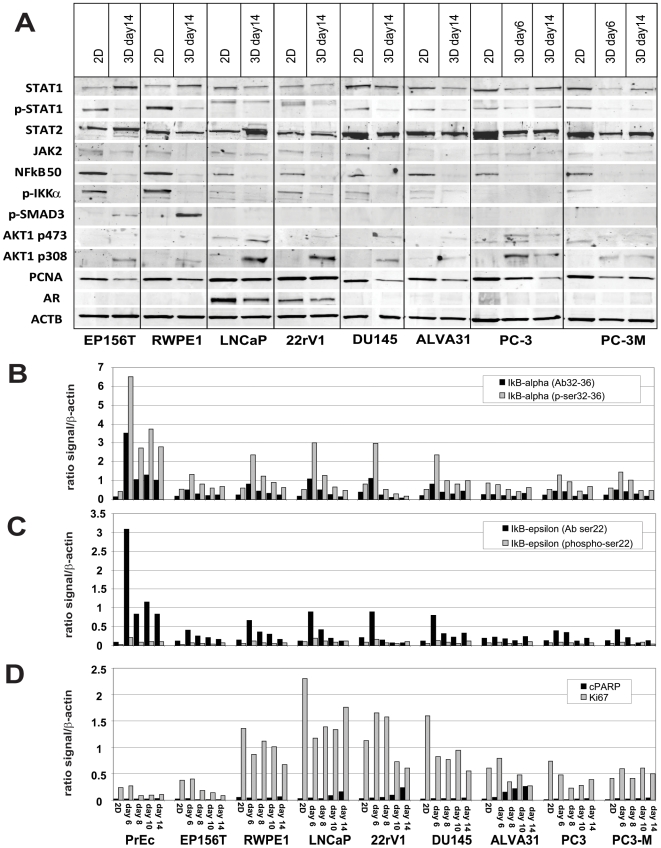
Altered expression of key signaling molecules, phospho-proteins and transcription factors in 2D versus 3D culture. A) Western blot analysis of various transcription factors (AKT1, STAT1, STAT2, IKKα, NFkB1 p50, SMAD3), androgen receptor (AR) and PCNA. B) and C) Protein lysate analysis of IkBα and IkBε (inhibitor of NF-kappaB) in cell lysates isolated from cells grown as monolayer in 2D or as spheroids in 3D culture. D) Lysate array analysis of proliferation marker Ki-67 and cleaved PARP, a marker for apoptosis. Relative ratio of specific antibody signal to overall β-actin signal was used for data normalization, allowing relative quantitative comparisons between cell lines, time points, and growth conditions.

The pathways most relevant for the formation of both round and mass spheroids in 3D were primarily related to lipid and steroid metabolism, prostaglandins/eicosanoids, and epigenetic regulation of gene expression. (Tables S5 and S6) Of the key signaling molecules identified, IGF1/IGF2 receptor, NFκB, pro-inflammatory chemokines (IL-1α, TNFα and CCL2), and AKT and PI3Kinase were suggested as the most prominent. The expression of NFκB1 (p50, p105); IKKα, STAT1 and p-STAT1 (but not STAT2); or Smad-3 were consistently reduced in spheroids compared to 2D (Figure 6a). This pattern is in agreement with temporarily increased levels of inhibitory IκBα and IκBε proteins (Figure 6b), peaking around days 6–8 of spheroid formation. This suggests the tight control of pro-inflammatory processes and chemokines/cytokines (e.g. CCL2) particularly at early stages of spheroid formation, but not in invasive structures. Lysate array analysis of phospo-GSK3β expression showed very similar dynamics (not shown), further supporting the temporary repression of both NFκB and Wnt signaling pathway during critical stages of spheroid formation.

#### Invasive/stellate phenotype

**Figure 7:**
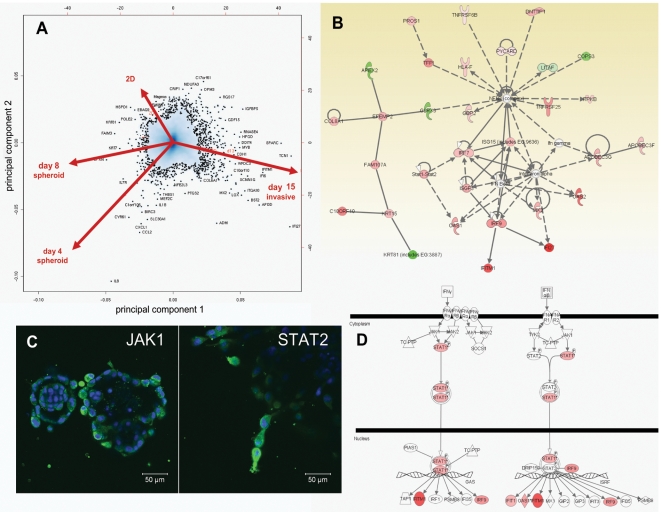
Functional analysis of dynamic gene expression changes observed in PC-3 cells, which correlate with the morphologic transformation of round to stellate spheroids. A) Principal component analysis PCA. Genes highlighted by arrows correlate best with PC-3 cells in monolayer (2D) culture, or round (day 4+ 8) and stellate phenotype (day 13+15) in 3D. B) IPA gene network analysis illustrating cross-talk between STAT1/STAT2, interferon α/β, and NFκB signaling in invasive cells (colors indicate factors of altered expression; red  =  induced, green  =  repressed). C) Immune staining of JAK1 and STAT2 protein expression in stellate PC- 3 spheroids. STAT2 signal is highest in invasive structures, JAK1 in peripheral cells. D) IPA: Schematic representation of Interferon γ and α/β signal transduction pathways, colors corresponding to fold changes of gene expression.

Core pathways identified in invasive cells (Figure 7a, Tables S5 and S6) were most prominently related to AKT and PI3Kinase, integrins, laminins, TGFβ, JAK/STAT & interferon signaling, hedgehog signaling, and matrix metalloproteinases (MMP13). Increased levels of pAKT1 compared to 2D conditions (both p-Ser308 and p-Ser473) were detected in most mass- and invasive-, but not in normal spheroids (Figure 6a). In invasive PC-3 cells, levels of these proteins were further increased. The expression of transcriptions factors STAT1 & STAT2, concomitant with interferon-inducible genes such as IFITM1 (interferon-inducible transmembrane protein 1), OAS1 or IFI27, point to the activation of JAK/STAT and interferon α/β-related signaling pathways in invasive cells (Figure 7a,b+d) as validated by immune fluorescence (Figure 7c) Since the expression of interferon-related genes and pathways was similar in both strongly branching RWPE-1 and invasive RWPE-2/w99, ALVA31, PC-3 or PC-3M cells, we postulate a general role of these mechanisms in cell motility.

### Compounds targeting AKT, PI3Kinase, and mTOR inhibit invasion in spheroid cells

**Figure 8:**
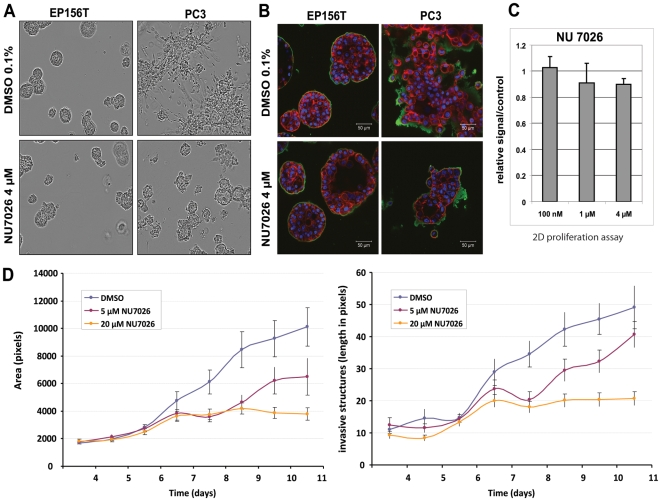
Exemplary response of spheroids from normal (EP156T) and invasive prostate cancer cells (PC-3) to the drug NU7026, a small molecule inhibitor specifically targeting PI3-kinase. A) Live-cell imaging of normal (EP156T) and invasive (PC-3) spheroids, treated with NU7027. B) Immune fluorescence of NU7026-treated EP156T and PC-3 spheroids, using an antibody targeting laminin β1 (green) and Alexa488-coupled phalloidin (F-actin). DAPI was used for nuclear staining. Drug exposure blocks the transformation of PC-3 spheroids into the stellate phenotype, but does not affect formation of normal spheroids. Vehicle-only (0.1% DMSO) served as a control. C) Lack of response of PC-3 cells to NU7026 (72 h) in monolayer culture (CTG, Cell Titer Glo assay). D) Quantitation of size (area, total # of pixels) and # of appendages (invasive structures, median length in pixels) in treated vs. untreated PC-3 spheroids. Bars represent the standard error.

Our miniaturized 3D culture system with a well-in-a-well microscopic format (15 experiments/plate), complemented with a high-content live-cell imaging system, and quantitative image analysis software, was developed for larger-scale compound testing in 3D. A library of >100 compounds was collected according to IPA, DrugBank, and Matador (Table S7), based on specific target genes or pathways/key signaling molecules suggested by Ingenuity pathway analysis. Compounds were first tested against “stellate” spheroids formed by PC3 and PC-3M cells, to identify inhibitors that may specifically block invasive tumor cells (Figure 8a+b). PC3 cells were also treated in monolayer culture (Figure 8c). Effective inhibitors identified were then further tested against a larger panel of cell lines in 3D, including non-transformed EP156T and RWPE-1 cells, and non-invasive DU145, LNCaP and 22rV1 cells (Figure S5). Small molecule inhibitors targeting PI3-Kinase (NU7026, APB2) and the AKT pathway (deguelin, API-2) most selectively inhibited invasion, proved less effective in 2D monolayer cultures,. The same inhibitors had only mild or no effects on normal cells. In contrast, most compounds targeting the mTOR and IGF1R pathways equally inhibited both invasive and non-invasive spheroids, normal cells in 3D, or cancer cells in monolayer cultures. Inhibitors against Hedgehog signaling (e.g. cyclopamine) also inhibited growth of both normal and cancer cells. In contrast, inhibitors targeting NFκB, pro-inflammatory chemokines & receptors (IL-1α and CCL2), TGFβ, p38 or p42/44MAP kinases were consistently ineffective against invasive and normal cells. Surprisingly, HDAC inhibitors and anti-mitotic drugs were ineffective, even at concentrations that were previously shown to cause apoptosis in monolayer culture [42].

## Discussion

We have characterized growth, differentiation and genome-wide mRNA expression patterns for a large panel of normal, non-transformed and prostate cell lines in Matrigel, covering all classic and many novel PrCa cell lines [43]. The development of miniaturized and cost-effective 3D models enabled us to monitor growth, maturation, invasion and motility of prostaspheres in real-time and high resolution, by combined live cell and confocal microscopy. These models will facilitate higher-throughput compound screens in 3D, allowing quantitative measurement of growth, size, shape, cellular dynamics and morphology of acinar structures. Recent research activities have mainly focused on the role of stem/progenitor cell populations in spheroids [39], [44], reviewed in [45]. With very few exceptions [46], these studies refer to prostaspheres cultured under anchorage-independent conditions, lacking any contact to ECM [47]. In contrast, our differentiation-related models showed essentially no enrichment of stem cell markers. It is clear and expected that lrECM primarily supports differentiation, but we were surprised that Matrigel is able to trigger normal-like epithelial differentiation programs even in PrCa cell lines that have been *in vitro* culture for over three decades. This essentially confirms the concepts formulated by Mina Bissell two decades ago, that context and in particular tumor environment matters and may powerfully override [48] malignant genotypes. However, our experimental data show that repression of the tumorigenic phenotype may also be only temporarily.

The specific aim of this study was a detailed analysis of various different modes of growth, migration and invasion of normal and prostate cancer cells, and the identification of small-molecule inhibitors that may specifically block invasive behavior. This is the first study describing the dynamic reversion of polarized epithelial spheroids into invasive cells, and gene co-expression networks associated with this transformation. While cell invasion and motility are traditionally analyzed by Boyden chamber, transwell or two-dimensional would-healing assays, our system provides a unique system to monitor and modulate invasive processes in an organotypic environment. Characterization of altered gene expression in spheroids and particularly invasive cells confirmed the importance of AKT and PI3-Kinase pathways in mammosphere- or prostasphere growth [39], [49]–[51]. However, AKT and PI3K pathways were shown to be particularly critical for invasion: Most drugs targeting these pathways effectively blocked aggressive invasion processes, but were less potent in 2D conditions, and often minimally affected growth and branching of normal cells. In contrast, mTOR, IGF1R and JAK/STAT pathways appeared to be primarily important for growth, branching and differentiation of both normal and tumor cells, regardless of the cell culture conditions, ECM and the microenvironment. Induction of JAK/STAT signaling, as reflected by the expression of many interferon-inducible proteins, may represent a general feature of migratory cells, and was observed in both branching and malignant invasive cells. Inflammation-related pathways [52] seemed less relevant for either growth or invasion. Compounds inhibiting the NFκB pathway were largely ineffective, in line with the observation of reduced expression of NFκB1, IKKα and increase of NFκB inhibitors IκBα, IκBε and IκBζ in maturing spheroids. Furthermore, although expression of pro-inflammatory chemokines (CCL2, IL-1α, TNFα) was induced in spheroid formation, compounds targeting the corresponding receptors proved ineffective. Most drugs inhibiting cell cycle progression/mitosis, p38 and p42/44 MAP kinases, or matrix metalloproteinase's (MMPs) were also ineffective against invasion, with the exception of WAY-170523, a specific inhibitor of MMP13 [53].

The pattern of invasion observed in aggressive PC-3 and PC-3M cells can be best described as streaming or chain migration [23], [24], and only occasionally single cells move by themselves. Invading cells transiently form and resolve cell-cell contacts, while moving along a common track through the ECM. The simultaneous induction of integrins such as ITGB2, ITGB4 and ITGA10, a panel of collagens and many other extracellular proteins indicates the importance of dynamic cell-matrix adhesion and attachment forces in this type of invasion. The over-expression of some of these markers in high grade PrCa may indicate that similar mechanisms and genes also play a role in vivo. Furthermore, dynamic actin polymerization-depolymerization cycles and Rho/Rac-mediated control of cell protrusion may be required for propelling migratory cells [33], [54]. Collective chain invasion is very different from the sheet- or tube-like movement observed in branching acinar morphogenesis of normal cells - a hallmark of normal organ development - and generally more dynamic. It is also different from amoeboid or gliding patterns of movement more commonly observed in 2D cultures.

The re-expression of epithelial markers such as laminin-5 [55], and the tight-junction protein Cx43 (GJP1) in invading cells is contradicting some previous reports in prostate [51], breast and ovarian cancers [9], [56], but it is consistent with the dynamic formation and resolution of cell-cell contacts in streaming invasion. Specific laminins may be required for lubrication and maintenance of tracks utilized as channels for invasion through the ECM. Guiding cells, referred to as “guerilla cells”, may provide overall orientation and direction (reviewed in [57]). The question whether fibroblasts may serve as guide cells remains to be elucidated [27]. In our models, guide cells can be identified by sharp, elongated and spindle-like filopodia, formed prior to the onset of invasion.

In addition to the re-expression of epithelial markers in invasive cells, streaming invasion is not considered a characteristic for mesenchymal cells or epithelial cells that have undergone an EMT. These are traditionally thought to migrate as single cells in a fibroblast-like fashion. Although an EMT genotype was indicated by the expression of mesenchymal markers, we were not able to define a clear mesenchymal, invasion-related phenotype. Furthermore, the invasive cells lacked prominent stem-cell related expression signatures [58] and did not acquire properties of CSCs (e.g. CD44+/CD24−). In contrast, expression of mesenchymal markers was a common feature in many cell lines and not causally related to malignant transformation nor invasiveness [59]. Mesenchymal markers are detected in branching (RWPE-1), round (DU145) and all stellate (RWPE-2/w99, ALVA31, PC-3, PC-3M), but not in mass-phenotype spheroids (e.g. LNCaP, 22rV1) with a prominent luminal phenotype. Round, early stage PC-3 and PC-3M spheroids expressed mesenchymal markers Vimentin and Fibronectin, which remained at the same expression levels even after the invasive conversion. Vimentin was co-expressed with epithelial markers such as cytokeratins 5 and 14 or E-cadherin in round spheroids, which did not interfere with epithelial polarization and differentiation (DU145, PC-3). Nuclear translocation of β-catenin and associated Wnt pathway induction, another hallmark of EMT [60], were not observed in invading cells. Of the classic E-box binding transcription factors associated with EMT, only expression of TWIST1 [61], [62] and ZEB1 correlated with the invasive potential of cell lines. None of these genes were further induced upon cell invasion. Surprisingly, Slug (SNAI2) expression was repressed during invasion, but strongly expressed in normal spheroids–suggesting a role in epithelial differentiation instead of EMT.

EMT as a developmental mechanism could be involved in normal developmental processes and invasive cancers alike, and likely represents a bidirectional process (EMT versus MET) [63]. In cancers, EMT might simply be a sign of increased tumor cell plasticity, rather than a key mechanism that provides invasive properties per se. Meta-stable and phenotypic flexible cancer cells, having undergone an EMT, are still capable of epithelial differentiation. This may be particularly relevant for the survival of micro-metastases in the blood stream, successful tissue colonization, and the formation of distant metastases [33]. It is interesting to note that despite the lack of both E-cadherin and alpha catenin, PC-3 cells are still able to form epithelial cell-cell contacts, apparently using alternative mechanisms which may not be a specialty restricted to this cell line. Further investigation of dynamic transformation of epithelial into invasive cells (and the reverse) may provide more general insights into these mechanisms, and the putative role of EMT. Recent reports confirm a possible function of EMT in mixed sheet- and chain migration patterns for various cell types [64], [65]. Expression of invasion-associated markers and pathways (AKT, PI3 kinase, STAT1), identified in our in vitro models, will be further investigated in clinical tumor samples, with a focus on high grade, metastasizing and invasive cancers.

In summary, our experimental systems facilitate the investigation of polarized epithelial structures or spheroids which mimic morphology, biochemistry, and invasive processes of tumors *in vitro*. We and others [26], [28] have shown that breast- and PrCa cell lines in 3D are representative for many questions relevant to tumor cell biology, rather poorly addressed in monolayer cell cultures. These 3D models can be useful and more reliable for cancer drug discovery and target identification, particularly if reproducibility and quantification of the relevant assays are properly addressed.

Our models provide comparatively low cost, high throughput *in vitro* tools for cancer research and drug discovery, allowing complex cell biology questions to be explored experimentally, and may partly reduce or replace animal xenograft models. 3D models could therefore serve as an intermediate decision-making step in the pre-clinical drug development pipeline, linking large scale high-throughput compound screens for lead identification and increasingly expensive validation studies based on animal xenografts.

## Supporting Information

Figure S1 Morphologically different multicellular structures are formed after embedding non-transformed/immortalized EP156T cells and PrCa cells (cell lines DU145 and PC-3) into purified collagen (left panel, PureCol; rat-tail collagen I), or growth-factor reduced Matrigel (right panel). Structures were imaged by phase-contrast microscopy (right side of each panel), and stained with Alexa488-conjugated phalloidin to highlight the cytoskeleton through F-actin (left side).
(6.22 MB TIF)

Figure S2 Representative confocal laser scanning images of spheroids formed in 3D Matrigel culture, stained with an antibody against laminins beta 1 (LAMB1) to highlight the formation of a basal lamina (BL) surrounding the structures formed in Matrigel. Round structures invariably have a complete, robust BL surrounding the entire spheroid. Mass phenotype spheroids have often thin, heterogeneous, and incomplete BL. Stellate structures show variable, often fuzzy BL structures, with a thin BL also surrounding the invasive cells. Grape-like structures do not have any recognizable BL. Single phenotype cells show spotty, irregular expression of laminins.
(7.45 MB TIF)

Figure S3 Analysis of markers and transcription factors related to epithelial-mesenchymal transition (EMT). A) Expression of epithelial-specific cadherin CDH1 (E-cadherin) versus mesenchymal-specific cadherin CDH2 (N-cadherin) across all cell lines, in monolayer and 3D culture. CDH2 is highly expressed in PC-3 and PC-3M, and co-expressed with CDH1 in RWPE-1 cells. B) Normalized gene expression values for a panel of epithelial- and mesenchymal-specific cadherins and EMT-related transcription factors in PrCa cell lines, as detected by Illumina bead arrays. C) Expression of CDH1 (E-cadherin) in spheroids formed by non-transformed, hTERT immortalized EP156T cells (strong and homogeneous), immortalized RWPE-1 cells (heterogeneous, strong in round structures but negative in branching acini), and PC-3 (negative).
(2.85 MB TIF)

Figure S4 Functional analysis of gene expression patterns, utilizing gene signatures associated with the six most closely related, prostate-cancer relevant pathways (AKT, PI3-kinase, IGF1 receptor, mTOR, S6 kinase, and PTEN). A) Composition of gene signatures, according to compilations by Biocompare (www.biocompare.com). B) Venn diagram, demonstrating overlaps between AKT, PI3-kinase, and mTOR pathway-associated genes. C) Heatmap, highlighting the expression of the most strongly invasion-related, up-regulated genes from combined pathway analyses in PC-3 cells, after transformation of round into stellate spheroids. D) Exemplary expression of collagen 1 subunit A1, in PrCa microarray samples analyzed through the expO gene expression consortium (www.intgen.org), indicating a positive association of expression with clinical parameters such as advanced stage, high grade tumors, and high Gleason score. The insert illustrates the relative expression of COL1A1 mRNA in normal prostate compared to prostate cancers (IST database, www.genesapiens.org).
(1.93 MB TIF)

Figure S5 Quantitative analysis of inhibitory drug effects on spheroid growth (area in pixels) for a panel of normal, non-transformed and cancer cell lines, using VTT ACCA image analysis software. Drugs, effective concentration, and major pathways inhibited by the compounds are indicated in the figure (text box).
(1.02 MB TIF)

Table S1 Cell lines and models used in this study.
(0.07 MB DOC)

Table S2 Antibodies used in this study.
(0.06 MB DOC)

Table S3 Summary of immune staining results for spheroids formed in 3D Matrigel culture.
(0.10 MB DOC)

Table S4 Gene Ontology Annotation for Gene Expression Clusters 1–12. Only the most significant enrichment factors and false discovery rates (FDR) are shown.
(0.18 MB DOC)

Table S5 Gene Set Enrichment Analysis GSEA, (A) for genes differentially expressed genes in monolayer vs. 3D spheroid culture in Matrigel, across all 10 cell lines analyzed, and (B) GSEA for differentially expressed genes in PC3 cells, comparing round (day 4+8) with stellate morphology (day 13+15).
(0.16 MB DOC)

Table S6 Ingenuity Pathway Analysis (IPA) for genes differentially expressed between 2D monolayer and 3D spheroid culture in Matrigel (general effects, 10 cell lines), and B) IPA for differentially expressed genes in PC3 cells, comparing round (day 4+8) with stellate morphology (day 13+15).
(0.06 MB DOC)

Table S7 Summary of small molecule inhibitors and drug treatments used in this study, directed against canonical pathways identified by functional gene expression analyses. Abbreviations: IB  =  invasion block; IAM  =  impaired acinar morphogenesis; GR  =  growth reduction; GA  =  growth arrest; CD  =  cell death.
(0.16 MB DOC)

Movie S1 Time lapse movie generated from live cell images (1 image/h), showing the formation of round spheroids by PC-3 cells. Movie sequence starts around day 8 after seeding into Matrigel. Round spheroids are then transformed into stellate structures, starting at approx. days 11–13 after inoculation.
(10.55 MB MP4)
