# Supplementary figures and images for: A Comprehensive Panel of Three-Dimensional Models for Studies of Prostate Cancer Growth, Invasion and Drug Responses
Source: PLoS One. 2010 May 3;5(5):e10431. doi: 10.1371/journal.pone.0010431 (PMC2862707; doi:10.1371/journal.pone.0010431)

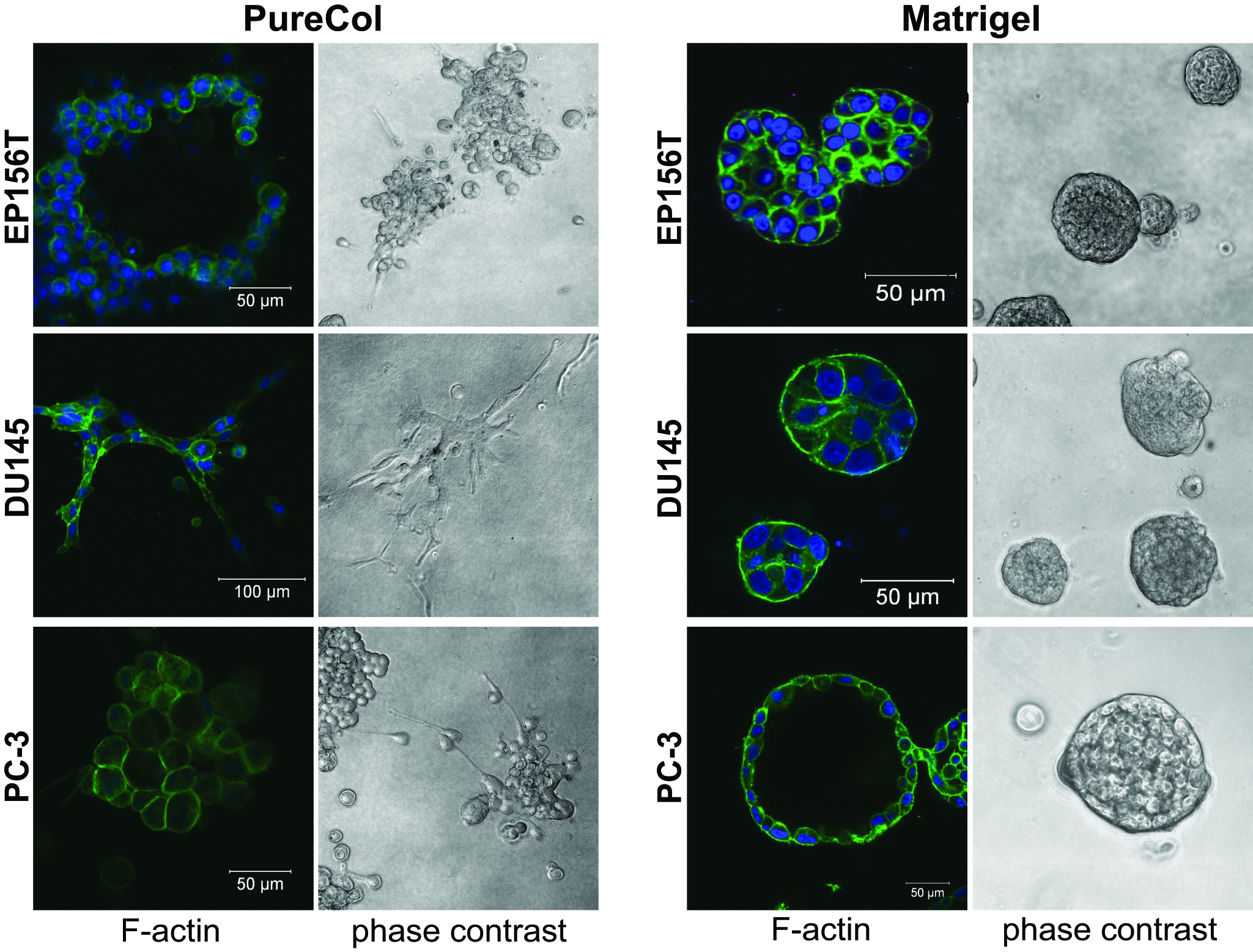

Supplement: Figure S1 — Morphologically different multicellular structures are formed after embedding non-transformed/immortalized EP156T cells and PrCa cells (cell lines DU145 and PC-3) into purified collagen (left panel, PureCol; rat-tail collagen I), or growth-factor reduced Matrigel (right panel). Structures were imaged by phase-contrast microscopy (right side of each panel), and stained with Alexa488-conjugated phalloidin to highlight the cytoskeleton through F-actin (left side). (6.22 MB TIF) [file pone.0010431.s001.tif]

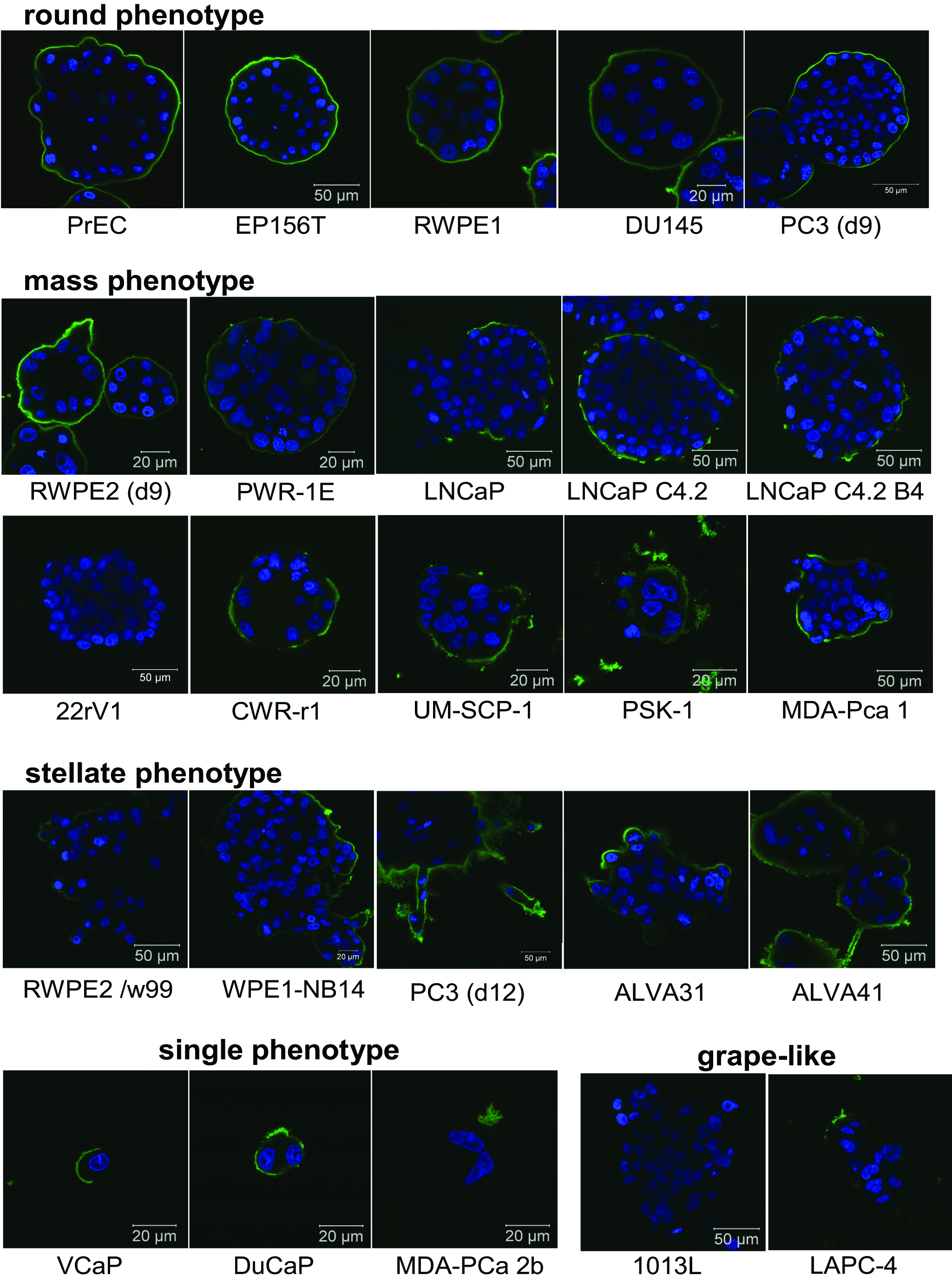

Supplement: Figure S2 — Representative confocal laser scanning images of spheroids formed in 3D Matrigel culture, stained with an antibody against laminins beta 1 (LAMB1) to highlight the formation of a basal lamina (BL) surrounding the structures formed in Matrigel. Round structures invariably have a complete, robust BL surrounding the entire spheroid. Mass phenotype spheroids have often thin, heterogeneous, and incomplete BL. Stellate structures show variable, often fuzzy BL structures, with a thin BL also surrounding the invasive cells. Grape-like structures do not have any recognizable BL. Single phenotype cells show spotty, irregular expression of laminins. (7.45 MB TIF) [file pone.0010431.s002.tif]

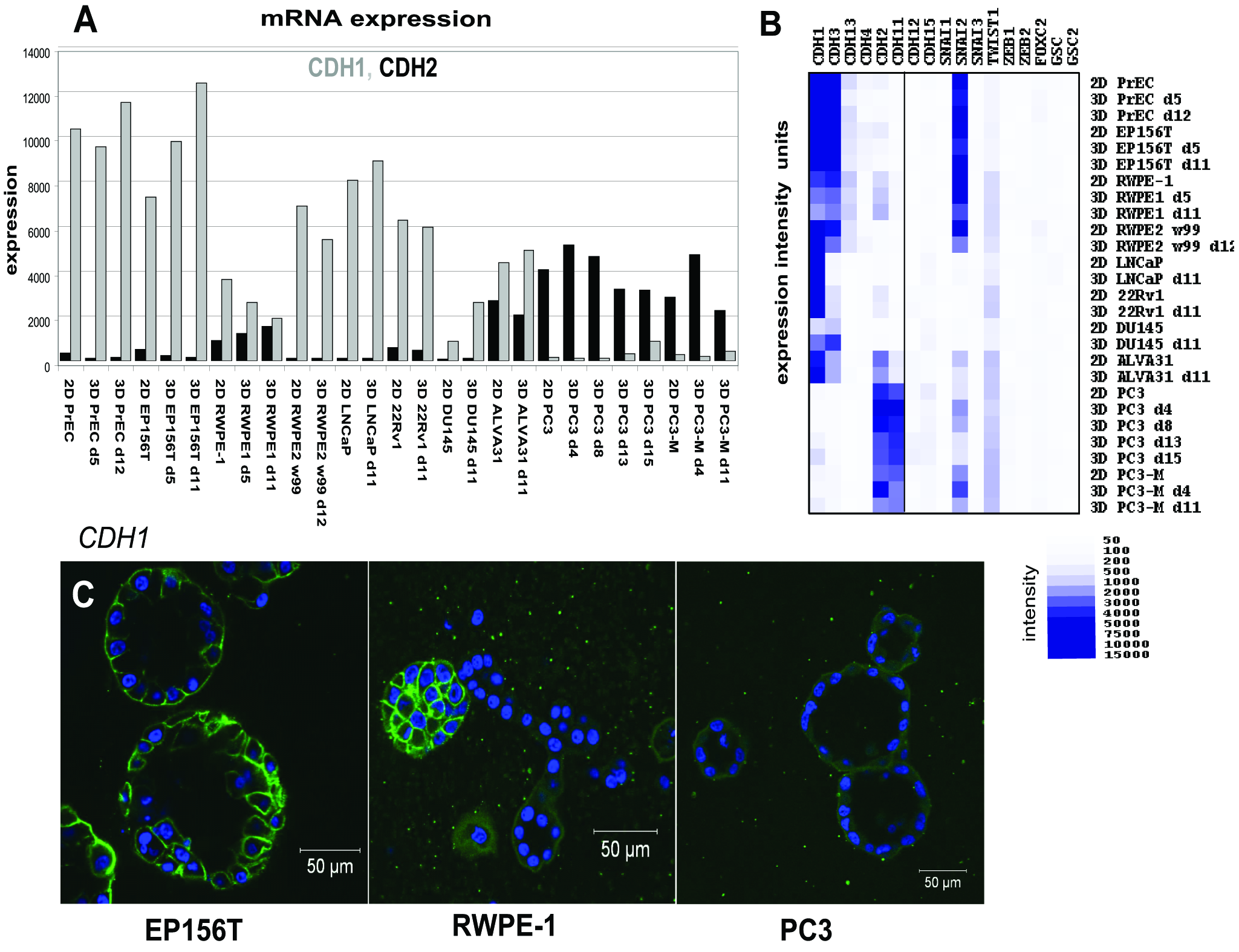

Supplement: Figure S3 — Analysis of markers and transcription factors related to epithelial-mesenchymal transition (EMT). A) Expression of epithelial-specific cadherin CDH1 (E-cadherin) versus mesenchymal-specific cadherin CDH2 (N-cadherin) across all cell lines, in monolayer and 3D culture. CDH2 is highly expressed in PC-3 and PC-3M, and co-expressed with CDH1 in RWPE-1 cells. B) Normalized gene expression values for a panel of epithelial- and mesenchymal-specific cadherins and EMT-related transcription factors in PrCa cell lines, as detected by Illumina bead arrays. C) Expression of CDH1 (E-cadherin) in spheroids formed by non-transformed, hTERT immortalized EP156T cells (strong and homogeneous), immortalized RWPE-1 cells (heterogeneous, strong in round structures but negative in branching acini), and PC-3 (negative). (2.85 MB TIF) [file pone.0010431.s003.tif]

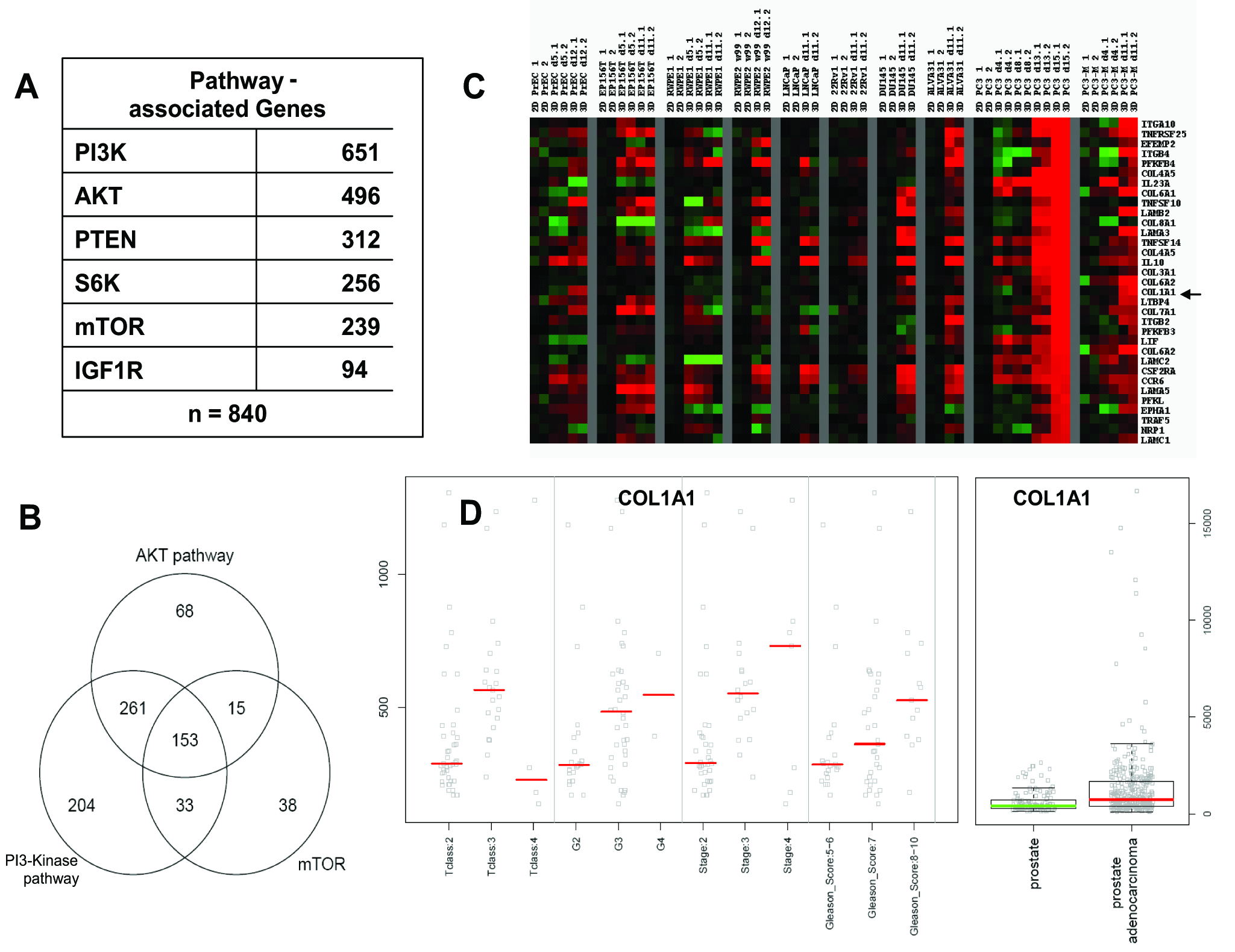

Supplement: Figure S4 — Functional analysis of gene expression patterns, utilizing gene signatures associated with the six most closely related, prostate-cancer relevant pathways (AKT, PI3-kinase, IGF1 receptor, mTOR, S6 kinase, and PTEN). A) Composition of gene signatures, according to compilations by Biocompare (www.biocompare.com). B) Venn diagram, demonstrating overlaps between AKT, PI3-kinase, and mTOR pathway-associated genes. C) Heatmap, highlighting the expression of the most strongly invasion-related, up-regulated genes from combined pathway analyses in PC-3 cells, after transformation of round into stellate spheroids. D) Exemplary expression of collagen 1 subunit A1, in PrCa microarray samples analyzed through the expO gene expression consortium (www.intgen.org), indicating a positive association of expression with clinical parameters such as advanced stage, high grade tumors, and high Gleason score. The insert illustrates the relative expression of COL1A1 mRNA in normal prostate compared to prostate cancers (IST database, www.genesapiens.org). (1.93 MB TIF) [file pone.0010431.s004.tif]

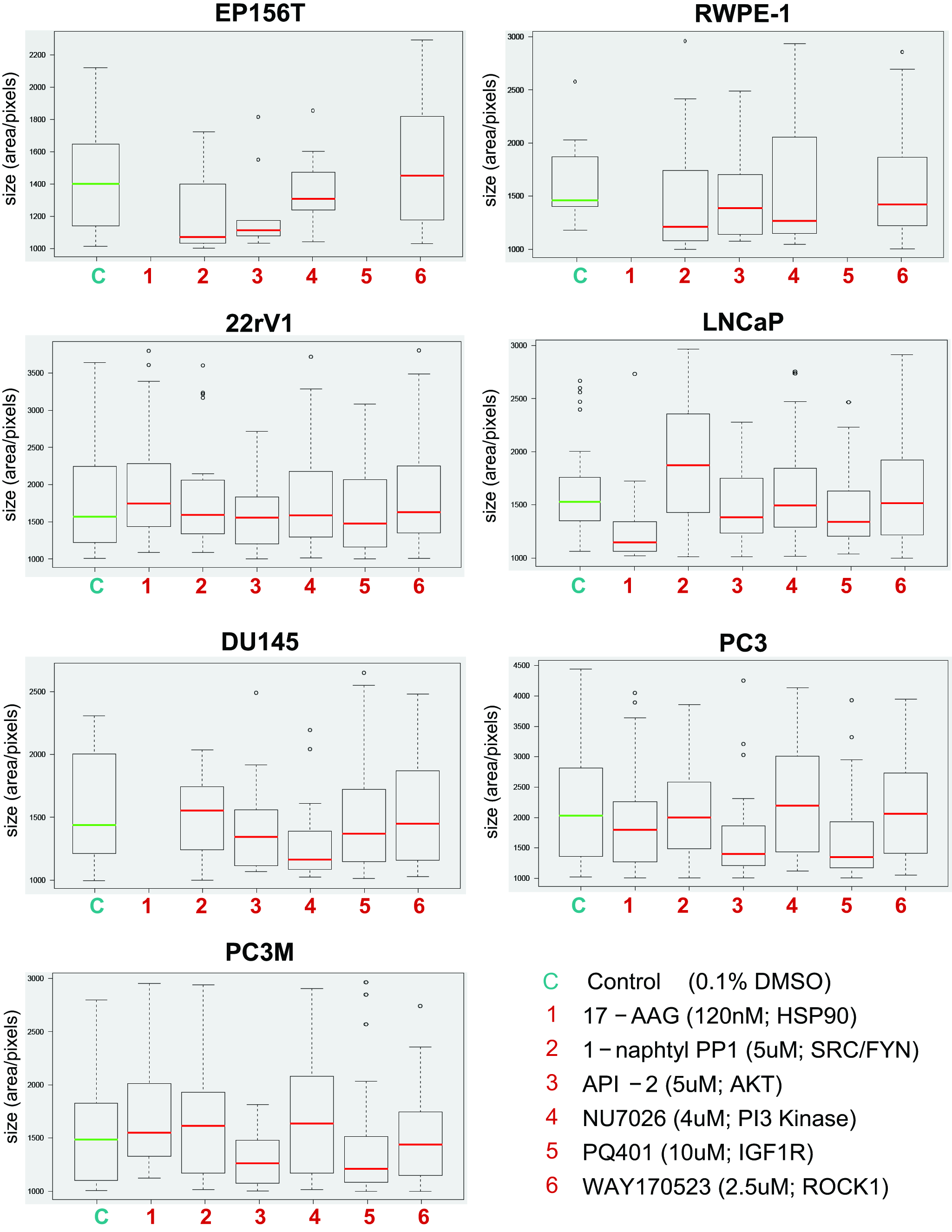

Supplement: Figure S5 — Quantitative analysis of inhibitory drug effects on spheroid growth (area in pixels) for a panel of normal, non-transformed and cancer cell lines, using VTT ACCA image analysis software. Drugs, effective concentration, and major pathways inhibited by the compounds are indicated in the figure (text box). (1.02 MB TIF) [file pone.0010431.s005.tif]
