# Supplementary material for: A Comprehensive Panel of Three-Dimensional Models for Studies of Prostate Cancer Growth, Invasion and Drug Responses
Source: PLoS One. 2010 May 3;5(5):e10431. doi: 10.1371/journal.pone.0010431 (PMC2862707; doi:10.1371/journal.pone.0010431)
Supplement: Table S1 — Cell lines and models used in this study. (0.07 MB DOC) [file pone.0010431.s006.doc]

**Table S1: Cell lines and models used in this study**

| **Line** | **Origin** | **Details** | **Source** | **Reference** |
| --- | --- | --- | --- | --- |
| **PrEC** | Normal epithelial | Prostate primary epithelial cells | Lonza (CC-2555) | - |
| **EP156T** | non-transformed epithelial | Immortalized with pBabe-hTERT-puro retroviral vector | Varda Rotter (Weizmann Institute of Science, Rehovot, Israel) | Kogan I, et al., Cancer Res. 2006 Apr ;66(7):3531-40. |
| **PWR-1E** | Normal epithelial | Immortalized with adenovirus type 12-SV40 hybrid (Ad12-SV40) | ATCC (CRL-11611) | Webber MM & Rhim JS.,US Patent 5,610,043 |
| **PZ-HPV-7** | non-transformed | non-transformed immortalized (HPV18) | ATCC (CRL-2221) | Weijerman PC et al., Cancer Res. 54, 5579 (1994) |
| **RWPE-1** | non-transformed epithelial | immortalized by human papilloma virus 18 (HPV-18) | ATCC (CRL-11609) | Bello D, et al., Carcinogenesis 18: 1215-1223, 1997. PMID 9214605 |
| **RWPE-2** | Transformed epithelial | RWPE-1 derivative, transformed by Kirsten murine sarcoma virus (Ki-MuSV) | ATCC (CRL-11610) | Bello D, et al., Carcinogenesis 18: 1215-1223, 1997. PMID 9214605 |
| **WPE1-NB14** | Transformed epithelial | RWPE-1 derivative transformed by N-methyl-N-nitrosourea (MNU) | ATCC (CRL-2850) | Bello D, et al., Carcinogenesis 18: 1215-1223, 1997. PMID 9214605 |
| **PC-3** | Adenocarcinoma | Bone metastasis | ATCC (CRL-1435) | Kaighn ME, et al., Invest. Urol. 17: 16-23, 1979. PMID 447482 |
| **PC3-M pro4** | Adenocarcinoma | highly metastatic variant of PC3 | Isaiah Fidler | Clinical Cancer Research 1996 2; 1627 |
| **DU145** | Adenocarcinoma | Brain metastasis | ATCC (HTB-81) | Stone KR, et al., Int. J. Cancer 21: 274-281, 1978. PMID 631930 |
| **LNCaP** | Adenocarcinoma | Left supraclavicular lymph node metastasis | ATCC (CRL-1740) | Horoszewicz JS, et al. LNCaP model of human prostatic carcinoma. Cancer Res. 43: 1809-1818, 1983. PMID 6831420 |
| **LNCaP C4-2** | Adenocarcinoma | Bone metastatic variant of LNCaP | Leland Chung (University of Berne, Switzerland) | Thalmann GN, et al., Prostate. 2000 Jul 1;44(2):91-103 Jul 1;44(2). |
| **LNCaP C4-2/B4** | Adenocarcinoma | Bone metastatic variant of LNCaP | Leland Chung (University of Berne, Switzerland) | Thalmann GN, et al., Prostate. 2000 Jul 1;44(2):91-103 Jul 1;44(2). |
| **CWR-R1** | Primary carcinoma | Androgen independent line derived from CWR22 xenograft model | Christopher Gregory (Univ. of North Carolina, Chapel Hill) | Moolky N, et al., Cancer Research, 56. 3042-3046. July 1. 1996 |
| **22Rv1** | Primary carcinoma | Androgen independent line derived from CWR22 xenograft model | ATCC (CRL-2505) | Sramkoski RM, et al., In Vitro Cell. Dev. Biol. Anim. 35: 403-409, 1999. PMID 10462204 |
| **CA-HPV-10** | Primary carcinoma | serum free tumor line | ATCC (CRL-2220) | Weijerman PC et al., Cancer Res. 54, 5579 (1994) |
| **MDA-PCa 1** | Primary carcinoma | Ascites metastasis | Nora Navone (Univ. of Texas, Houston, TX) | Navone NM, et al., Clin. Cancer Res. 3: 2493-2500, 1997. PMID 9815652 |
| **UM-SCP-1** | Primary squamous cell carcinoma | Squamous cell carcinoma of the prostate | Barton Grossman (Univ. of Texas, Houston, TX) | Grossman HB, et al., Cancer Research 44, 4111 -4117, 1984 |

| **Line** | **Origin** | **Details** | **Source** | **Reference** |
| --- | --- | --- | --- | --- |
| **LAPC-4** | Primary transitional cell carcinoma | lymph node metastasis of xenografted mouse | Charles Sawyer (Univ. of California, Los Angeles, CA) | Klein KA, et al., Nat Med 1997;3:402–408. |
| **ALVA31** | Adenocarcinoma | likely PC3 derivative | Department of Veterans Affairs Medical Center, Tacoma, WA | Mehta PP, et al., Mol Carcinog. 1996 Jan;15(1):18-32. |
| **ALVA41** | Adenocarcinoma | likely PC3 derivative | Department of Medicine, St. Luke's-Roosevelt Hospital Center, Columbia University, New York, NY | Mehta PP, et al., Mol Carcinog. 1996 Jan;15(1):18-32. |
| **RWPE-2/w99** | Transformed epithelial | RWPE-1 derivative cloned in soft agar to select cells that show high expression of Ki-ras | ATCC (CRL-2853) | Bello D, et al., Carcinogenesis 18: 1215-1223, 1997. PMID 9214605 |
| **1013L** | Primary transitional cell carcinoma | derived from xenograft model | Anita Bilström (Active Biotech Research AB, Lund, Sweden) | Williams RD., Invest Urol 1980;17:359–363. |
| **VCaP** | Adenocarcinoma | Vertebral metastasis | ATCC (CRL-2876) | Korenchuk S, et al.,In Vivo 15: 163-168, 2001. PMID 11317522 |
| **DuCaP** | Adenocarcinoma | Dura mater metastasis | Kenneth Pienta (Univ. of Michigan, Ann Arbor, MI) | Lee YG, et al., In Vivo. 2001 Mar-Apr;15(2):157-62. |
| **NCI-H660** | Adenocarcinoma | lung metastasis | ATCC (CRL-5813) | AF Gazdar ; J. Minna (1989) |
| **MDA PCa 2b** | Adenocarcinoma | Bone metastasis | ATCC (CRL-2422) | Navone NM, et al., Clin. Cancer Res. 3: 2493-2500, 1997. PMID 9815652 |
| **PSK-1** | Prostatic small-cell carcinoma |  | Chol Jang Kim (Shiga Univ. of Medical Science, Ottsu, Japan) | Kim CJ, et al., Prostate. 2000 Mar 1;42(4):287-94. |
