# Supplementary material for: A Comprehensive Panel of Three-Dimensional Models for Studies of Prostate Cancer Growth, Invasion and Drug Responses
Source: PLoS One. 2010 May 3;5(5):e10431. doi: 10.1371/journal.pone.0010431 (PMC2862707; doi:10.1371/journal.pone.0010431)
Supplement: Table S2 — Antibodies used in this study. (0.06 MB DOC) [file pone.0010431.s007.doc]

**Table S2: Antibodies used in this study**

| **Antibody** | **Manufacturer** | **Catalog number** |
| --- | --- | --- |
| Anti-Androgen Receptor Ab-1 (Clone AR 441) | Labvision | MS-443 |
| Anti-Beta-actin | Abcam | ab8226 |
| Anti-CD44 | Abcam | ab19622 |
| Anti-Chromogranin A | Abcam | ab715 |
| Anti-Cleaved PARP (Asp214) (19F4) | Cell signaling | 9546S |
| Anti-Cytokeratin 14 | Abcam | ab7800 |
| Anti-Cytokeratin 18 | Abcam | ab32118 |
| Anti-Cytokeratin 8 | Abcam | ab14053 |
| Anti-GM130 | Abcam | ab40881 |
| Anti-IKKα (Phospho-Thr23) | Full Moon Biosystems | 65450 |
| Anti-Integrin β-1 | Abcam | ab7168 |
| Anti-active Integrin β-1 [12G10] | Abcam | ab30394 |
| Anti-IκB-α (Ab-32/36) | Full Moon Biosystems | 75443 |
| Anti-IκB-α (Phospho-Ser32/Ser36) | Full Moon Biosystems | 65473 |
| Anti-IκB-ε (Ab-Ser22) | Full Moon Biosystems | 75636 |
| Anti-IκB-ε (Phospho-Ser22) | Full Moon Biosystems | 65636 |
| Anti-Jak2 (D2E12) | Cell signaling | 3230 |
| Anti-Ki67 | Abcam | ab15580 |
| Anti-Laminin α-1 | Santa Cruz | sc-59849 |
| Anti-Laminin (alpha/beta) LAM-89 | Abcam | ab49726 |
| Anti-Laminin β-1 | Abcam | ab44941 |
| Anti-NF-κB p105/p50 | Full Moon Biosystems | 75338 |
| Anti-p63 | Abcam | ab735 |
| Anti-PCNA antibody [PC10] | Abcam | ab29 |
| Anti-Phospho-Akt (Ser473) | Cell signaling | CST9271S |
| Anti-Phospho-Akt (Thr308) | Cell signaling | 9275 |
| Anti-Phospho-Smad3 (Ser423/425) (C25A9) | Cell signaling | 9520 |
| Anti-Phospho-Stat1 (Tyr701) | Cell signaling | 9171 |
| Anti-Prostate Specific Antigen PSA (KLK3) | Abcam | ab9537 |
| Anti-Stat1 | Cell signaling | 9172 |
| Anti-Stat2 | Sigma Aldrich | HPA018888 |
