# Supplementary material for: A Comprehensive Panel of Three-Dimensional Models for Studies of Prostate Cancer Growth, Invasion and Drug Responses
Source: PLoS One. 2010 May 3;5(5):e10431. doi: 10.1371/journal.pone.0010431 (PMC2862707; doi:10.1371/journal.pone.0010431)
Supplement: Table S3 — Summary of immune staining results for spheroids formed in 3D Matrigel culture. (0.10 MB DOC) [file pone.0010431.s008.doc]

**Table S3: Summary of immune staining results for spheroids grown in matrigel, from the indicated cell lines.**

| Group | Cell line | LAMB1 | CK8 | CK18 | CK14 | ITGB1 | AR | PSA | CHGA | CD44 |
| --- | --- | --- | --- | --- | --- | --- | --- | --- | --- | --- |
| round | **PrEC (branching)** | C | +++ | - | +++ / - | +++ | - | - | +++ | +++ |
|  | **RWPE-1 (branching)** | C | +++ | - | +++ / - | +++ | - | - | +++ | +++ |
|  | **EP156T** | C | +++ | - | +++ | +++ | - | - | +++ | +++ |
|  | **PC-3**** (day 1-9) | C | +++ | - | + | +++ | - | - | ++ | +++ |
|  | **DU145** | C | +++ | - | + | +++ | - | - | ++ | +++ |
| mass | **PWR-1E (branching)** | C | +++ | - | +++ / - | +++ | - | - | - | +++ |
|  | **RWPE-2*** | C / D | - | - | +++ | +++ | - | - | +++ | +++ |
|  | **WPE1-NB14*** | C / D | +++ | - | +++ | +++ | - | - | + | +++ |
|  | **LNCaP** | D | - | ++ | ++ | +++ | N / CP | + | + | +++ |
|  | **LNCaP C4-2** | D | ++ | - | +++ | +++ | N / CP | +++ | - | + |
|  | **LNCaP C4-2B** | D | ++ | - | +++ | +++ | N / CP | +++ | - | +++ |
|  | **CWR-R1** | D | +++ | + | ++ | - | N / CP | +++ | - | +++ |
|  | **22Rv1** | D | ++ | - | ++ | - | CP | + | +++ | +++ |
|  | **MDA PCa 1** | D | +++ | - | ++ | +++ | CP | - | ++ | +++ |
|  | **UM-SCP-1** | D | ++ | - | ++ | +++ | - | - | ++ | +++ |
| grape-like | **LAPC-4** | - | +++ | - | ++ | +++ | CP | - | ++ | +++ |
|  | **1013L** | - | - | +++ | +++ | ++ (CP) | - | - | + | +++ |
| single | **VCaP** | + | +++ | + | +++ | +++ | N / CP | +++ | + | + |
|  | **DuCaP** | + | +++ | - | +++ | +++ | CP | +++ | - | ++ |
|  | **MDA PCa 2b** | - | +++ | + | +++ | +++ | CP | +++ | + | +++ |
| stellate | **PC3** (after day 11) | D | ++ | - | + | ++ | - | - | - | +++ |
|  | **PC3-M** | D | ++ | - | + | ++ | - | - | - | +++ |
|  | **ALVA31** | D | ++ | - | + | ++ | - | - | - | +++ |
|  | **ALVA41** | D | ++ | + | + | +++ | - | - | ++ | +++ |
|  | **RWPE-2/w99** | D | ++ | - | +++ | +++ | - | - | + | +++ |

- = Negative, + = Weak staining, ++ = Moderate staining, +++ = Strong staining, C = Continuous, D = Disrupted, CP = Cytoplasmic, N = Nuclear (can also be cytoplasmic); * heterogeneous phenotype, ** phenotypic switch round-stellate
