# Supplementary material for: A Comprehensive Panel of Three-Dimensional Models for Studies of Prostate Cancer Growth, Invasion and Drug Responses
Source: PLoS One. 2010 May 3;5(5):e10431. doi: 10.1371/journal.pone.0010431 (PMC2862707; doi:10.1371/journal.pone.0010431)
Supplement: Table S4 — Gene Ontology Annotation for Gene Expression Clusters 1–12. Only the most significant enrichment factors and false discovery rates (FDR) are shown. (0.18 MB DOC) [file pone.0010431.s009.doc]

**Table S4: Gene Ontology Annotation for Clusters 1-12**

| **Cl** | **Gene Ontology Term** | **PValue** | **FDR** | **#** |
| --- | --- | --- | --- | --- |
| **1** | **Extracellular Matrix Turnover** |  |  |  |
|  | GO:0005576 extracellular region | 2.07E-07 | 1.79E-04 | 43 |
|  | GO:0005615 extracellular space | 7.48E-06 | 2.16E-03 | 22 |
|  | **Lipid/Steroid Metabolism** |  |  |  |
|  | GO:0006690 icosanoid metabolic process | 2.50E-05 | 6.35E-02 | 7 |
|  | GO:0006629 lipid metabolic process | 5.08E-05 | 8.50E-02 | 26 |
|  | GO:0006693 prostaglandin metabolic process | 7.16E-05 | 6.08E-02 | 5 |
|  | **Oxidoreductases** |  |  |  |
|  | GO:0004033 aldo-keto reductase activity | 4.06E-04 | 2.53E-01 | 4 |
|  | GO:0016614 oxidoreductase activity | 1.82E-04 | 1.61E-01 | 9 |
|  | GO:0016616 oxidoreductase activity | 9.55E-05 | 1.28E-01 | 9 |
|  | **Development/Cell Differentiation** |  |  |  |
|  | GO:0048513 organ development | 6.62E-05 | 6.72E-02 | 36 |
|  | GO:0009653 morphogenesis | 7.53E-05 | 4.83E-02 | 33 |
|  | GO:0048856 anatomical structure development | 2.02E-04 | 8.46E-02 | 50 |

|  | **Development/Cell Differentiation** | **PValue** | **FDR** | **#** |
| --- | --- | --- | --- | --- |
| **2** | GO:0048856 anatomical development | 1.56E-06 | 8.15E-03 | 47 |
|  | GO:0009653 morphogenesis | 2.67E-06 | 6.99E-03 | 31 |
|  | GO:0032502 developmental process | 3.42E-06 | 5.98E-03 | 61 |
|  | **Epithelial Cell Differentiation** |  |  |  |
|  | GO:0008544 epidermis development | 1.81E-04 | 1.12E-01 | 9 |
|  | GO:0009888 tissue development | 2.71E-04 | 1.46E-01 | 13 |
|  | GO:0007398 ectoderm development | 2.96E-04 | 1.44E-01 | 9 |

|  | **Lipid/Steroid Metabolism** | **PValue** | **FDR** | **#** |
| --- | --- | --- | --- | --- |
| **6** | GO:0016125 sterol metabolic process | 3.25E-11 | 1.71E-07 | 17 |
|  | GO:0008203 cholesterol metabolic process | 5.52E-10 | 1.45E-06 | 15 |
|  | GO:0016126 sterol biosynthetic process | 1.27E-09 | 2.23E-06 | 11 |
|  | GO:0008202 steroid metabolic process | 2.74E-09 | 3.60E-06 | 21 |
|  | GO:0006066 alcohol metabolic process | 2.16E-08 | 2.27E-05 | 26 |
|  | **Epigenetic Regulation** |  |  |  |
|  | GO:0016568 chromatin modification | 9.56E-05 | 3.52E-02 | 15 |
|  | GO:0051276 chromosome organization | 2.76E-04 | 8.68E-02 | 21 |

|  | **Gene Ontology Term** | **PValue** | **FDR** | **#** |
| --- | --- | --- | --- | --- |
| **7** | **Mitochondrion** |  |  |  |
|  | GO:0005739 mitochondrion | 1.22E-07 | 8.12E-06 | 38 |
|  | **Ribosome** |  |  |  |
|  | GO:0042254 ribosome biogenesis | 1.85E-07 | 9.70E-04 | 12 |
|  | GO:0022613 ribonucleoprotein assembly | 4.16E-06 | 1.09E-02 | 15 |
|  | GO:0006364 rRNA processing | 5.02E-04 | 2.81E-01 | 7 |
|  | **Ribosomal RNA processing** |  |  |  |
|  | GO:0005730 nucleolus | 1.34E-06 | 6.45E-05 | 14 |
|  | GO:0005732 small nucleolar ribonucleoprotein | 1.94E-06 | 8.42E-05 | 6 |
|  | **General Metabolism** |  |  |  |
|  | GO:0043170 macromolecule metabolic process | 9.19E-05 | 1.14E-01 | 144 |
|  | GO:0044237 cellular metabolic process | 1.06E-04 | 1.05E-01 | 160 |
|  | GO:0008152 metabolic process | 2.55E-04 | 1.74E-01 | 172 |

|  | **Gene Ontology Term** | **PValue** | **FDR** | **#** |
| --- | --- | --- | --- | --- |
| **8** | **Cell Cycle/Mitosis** |  |  |  |
|  | GO:0022402 cell cycle process | 1.94E-57 | 1.02E-53 | 91 |
|  | GO:0000279 M phase | 2.69E-57 | 7.05E-54 | 65 |
|  | GO:0007049 cell cycle | 4.41E-56 | 7.72E-53 | 96 |
|  | **Mitosis/Chromosome** |  |  |  |
|  | GO:0005694 chromosome | 5.08E-25 | 2.20E-22 | 43 |
|  | GO:0000775 chromosome, pericentric region | 4.25E-19 | 4.61E-17 | 20 |
|  | **Mitosis/Spindle Apparatus** |  |  |  |
|  | GO:0015630 microtubule cytoskeleton | 3.46E-22 | 5.01E-20 | 41 |
|  | GO:0005819 spindle | 2.49E-21 | 3.09E-19 | 22 |
|  | GO:0005815 microtubule organizing center | 1.84E-13 | 8.86E-12 | 20 |
|  | **Nucleotide & DNA synthesis** |  |  |  |
|  | GO:0005524 ATP binding | 6.44E-17 | 3.20E-13 | 68 |
|  | GO:0032559 adenyl ribonucleotide binding | 1.19E-16 | 1.60E-13 | 68 |
|  | GO:0030554 adenyl nucleotide binding | 1.97E-15 | 1.92E-12 | 68 |
|  | GO:0032555 purine ribonucleotide binding | 4.17E-13 | 3.00E-10 | 70 |

|  | **Gene Ontology Term** | **PValue** | **FDR** | **#** |
| --- | --- | --- | --- | --- |
| **9** | **Cell Differentiation** |  |  |  |
|  | GO:0048513 organ development | 2.06E-12 | 3.60E-09 | 31 |
|  | GO:0032502 developmental process | 1.61E-11 | 2.11E-08 | 47 |
|  | GO:0048731 system development | 4.62E-11 | 4.85E-08 | 34 |
|  | **Development & Apoptosis** |  |  |  |
|  | GO:0065007 biological regulation | 6.19E-11 | 5.42E-08 | 59 |
|  | GO:0043066 negative regulation of apoptosis | 4.89E-07 | 1.03E-04 | 11 |
|  | GO:0043069 negative regulation of cell death | 5.50E-07 | 1.11E-04 | 11 |
|  | GO:0048468 cell development | 2.36E-06 | 3.54E-04 | 22 |
|  | **Extracellular Matrix Turnover** |  |  |  |
|  | GO:0044421 extracellular region part | 6.40E-07 | 5.56E-04 | 19 |
|  | GO:0005576 extracellular region | 1.23E-06 | 5.32E-04 | 24 |
|  | **Cell Proliferation** |  |  |  |
|  | GO:0008283 cell proliferation | 7.12E-09 | 2.88E-06 | 21 |
|  | GO:0042127 regulation of cell proliferation | 7.24E-08 | 2.11E-05 | 16 |
|  | **Stress & Immune Response/Chemokines** |  |  |  |
|  | GO:0006950 response to stress | 1.83E-09 | 1.07E-06 | 25 |
|  | GO:0002376 immune system process | 8.01E-09 | 3.01E-06 | 25 |
|  | GO:0009605 response to external stimulus | 9.03E-09 | 3.16E-06 | 19 |
|  | GO:0009611 response to wounding | 1.04E-08 | 3.42E-06 | 16 |
|  | GO:0006955 immune response | 1.09E-07 | 2.73E-05 | 21 |
|  | GO:0042330 taxis | 1.18E-07 | 2.83E-05 | 10 |
|  | GO:0006935 chemotaxis | 1.18E-07 | 2.83E-05 | 10 |
|  | GO:0005125 cytokine activity | 1.91E-07 | 2.75E-04 | 12 |
|  | GO:0008009 chemokine activity | 9.00E-06 | 6.46E-03 | 6 |

|  | **Gene Ontology Term** | **PValue** | **FDR** | **#** |
| --- | --- | --- | --- | --- |
| **10** | **Cell Adhesion** |  |  |  |
|  | GO:0022610 biological adhesion | 4.66E-06 | 2.42E-02 | 32 |
|  | GO:0007155 cell adhesion | 4.66E-06 | 2.42E-02 | 32 |
|  | **Extracellular Matrix Turnover** | 4.54E-05 |  |  |
|  | GO:0044420 extracellular matrix part | 7.44E-11 | 6.46E-08 | 17 |
|  | GO:0005578 proteinaceous extracellular matrix | 1.87E-08 | 8.11E-06 | 24 |
|  | GO:0005581 collagen | 2.53E-08 | 7.31E-06 | 10 |
|  | GO:0031012 extracellular matrix | 2.60E-08 | 5.64E-06 | 24 |
|  | **Development/Cell Differentiation** |  |  |  |
|  | GO:0048856 anatomical structure development | 9.10E-05 | 9.12E-02 | 59 |
|  | GO:0032501 multicellular organismal process | 2.66E-04 | 1.60E-01 | 88 |
|  | GO:0048731 system development | 3.19E-04 | 1.70E-01 | 49 |

|  | **Gene Ontology Term** | **PValue** | **FDR** | **#** |
| --- | --- | --- | --- | --- |
| **11** | **Interferons/Chemokines** |  |  |  |
|  | GO:0009615 response to virus | 9.63E-18 | 5.06E-14 | 16 |
|  | GO:0009607 response to biotic stimulus | 1.09E-14 | 2.86E-11 | 20 |
|  | GO:0051707 response to other organism | 2.57E-14 | 3.37E-11 | 17 |
|  | **Immune Response** |  |  |  |
|  | GO:0006955 immune response | 1.90E-14 | 3.32E-11 | 30 |
|  | GO:0002376 immune system process | 1.05E-13 | 1.10E-10 | 32 |
|  | GO:0050896 response to stimulus | 1.46E-11 | 1.28E-08 | 47 |

|  | **Gene Ontology Term** | **PValue** | **FDR** | **#** |
| --- | --- | --- | --- | --- |
| **12** | **Protein Kinase Regulation** |  |  |  |
|  | GO:0019210 kinase inhibitor activity | 12.12144 | 1.23E-04 | 6 |
|  | GO:0004857 enzyme inhibitor activity | 3.147322 | 0.001577 | 12 |
