# Supplementary material for: A Comprehensive Panel of Three-Dimensional Models for Studies of Prostate Cancer Growth, Invasion and Drug Responses
Source: PLoS One. 2010 May 3;5(5):e10431. doi: 10.1371/journal.pone.0010431 (PMC2862707; doi:10.1371/journal.pone.0010431)
Supplement: Table S5 — Gene Set Enrichment Analysis GSEA, (A) for genes differentially expressed genes in monolayer vs. 3D spheroid culture in Matrigel, across all 10 cell lines analyzed, and (B) GSEA for differentially expressed genes in PC3 cells, comparing round (day 4+8) with stellate morphology (day 13+15). (0.16 MB DOC) [file pone.0010431.s010.doc]

**Table S5: A. Gene Set Enrichment Analysis, genes differentially expressed between 2D and 3D Matrigel culture (all 10 cell lines).**

| ***#*** | ***Gene set name*** | ***Score*** | ***p-value*** | ***FDR*** | ***category*** |
| --- | --- | --- | --- | --- | --- |
| 570 | **GA13 PATHWAY** | 0.7546 | 0 | 0 | canonical |
| 260 | **HISTONE MODIFICATION** | 0.634 | 0 | 0 | GO |
| 142 | **COVALENT CHROMATIN MODIFICATION** | 0.5855 | 0 | 0 | GO |
| 66 | **CCR3 PATHWAY** | 0.5308 | 0 | 0 | canonical |
| 770 | **STEROID BIOSYNTHETIC PROCESS** | 0.5116 | 0 | 0 | GO |
| 297 | **XBP1** | 0.413 | 0 | 0 | TF |
| 527 | **MYOGNF1** | 0.3994 | 0.01 | 0.3793 | TF |
| 125 | **NFKB (GGGNNTTTCC)** | 0.3593 | 0.01 | 0.3793 | TF |
| 439 | **MAZR** | 0.3264 | 0 | 0 | TF |
| 458 | **CAC BINDING PROTEIN** | 0.286 | 0 | 0 | TF |
| 288 | **IRF2** | 0.2749 | 0.01 | 0.3793 | TF |
| 363 | **CP2** | 0.2221 | 0 | 0 | TF |
| 71 | **SREBP1 (TCANNTGAY)** | 0.1939 | 0.01 | 0.3793 | TF |
| 553 | **TFIIA** | 0.1901 | 0 | 0 | TF |
| 238 | **AP1** | 0.1859 | 0.01 | 0.3793 | TF |
| 337 | **JAK/STAT SIGNALING PATHWAY** | 0.1791 | 0 | 0 | canonical |
| **negative scores:** | | | | | |
| 461 | **NUCLEOTIDE METABOLISM** | -1.249 | 0.02 | 0.4711 | canonical |
| 19 | **AMINOACYL TRNA BIOSYNTHESIS** | -1.0797 | 0.02 | 0.4711 | canonical |
| 174 | **DNA DEPENDENT DNA REPLICATION** | -1.0797 | 0 | 0 | GO |
| 74 | **CELL CYCLE (KEGG)** | -1.0665 | 0 | 0 | canonical |
| 320 | **M PHASE OF MITOTIC CELL CYCLE** | -0.9796 | 0 | 0 | GO |
| 36 | **ATR/BRCA PATHWAY** | -0.9654 | 0.01 | 0.3533 | canonical |
| 349 | **MITOSIS** | -0.9615 | 0 | 0 | GO |
| 352 | **MITOTIC SISTER CHROMATID SEGREGATION** | -0.9613 | 0 | 0 | GO |
| 291 | **AMINOACYL TRNA BIOSYNTHESIS** | -0.9549 | 0.02 | 0.4711 | canonical |
| 525 | **RNA TRANSCRIPTION REACTOME** | -0.9421 | 0 | 0 | canonical |
| 313 | **CELL CYCLE (HSA04110)** | -0.933 | 0 | 0 | canonical |
| 647 | **REGULATION OF MITOSIS** | -0.927 | 0 | 0 | GO |
| 464 | **ONE CARBON POOL BY FOLATE** | -0.9269 | 0 | 0 | canonical |
| 303 | **DNA POLYMERASE (HSA03030)** | -0.9153 | 0.01 | 0.3533 | canonical |
| 176 | **DNA INTEGRITY CHECKPOINT** | -0.9044 | 0 | 0 | GO |
| 756 | **SISTER CHROMATID SEGREGATION** | -0.8732 | 0 | 0 | GO |

**B. GSEA for differentially expressed genes in PC3 cells, comparing round (day 4 and 8) with stellate morphology (day 13 + 15).**

| ***#*** | ***Gene set name*** | ***Score*** | ***p-value*** | ***FDR*** | ***category*** |
| --- | --- | --- | --- | --- | --- |
| 397 | **NEGATIVE REGULATION OF MAP KINASE ACTIVITY** | 0.9589 | 0 | 0 | GO |
| 309 | **LIPID HOMEOSTASIS** | 0.761 | 0 | 0 | GO |
| 328 | **ECM RECEPTOR INTERACTION HSA04512** | 0.6481 | 0.001 | 0.1413 | canonical |
| 306 | **LEUKOCYTE MIGRATION** | 0.63 | 0.012 | 0.4462 | GO |
| 268 | **ICOSANOID METABOLIC PROCESS** | 0.6227 | 0 | 0 | GO |
| 97 | **CELLULAR CARBOHYDRATE CATABOLIC PROCESS** | 0.5865 | 0 | 0 | GO |
| 414 | **LAIR PATHWAY** | 0.5637 | 0.001 | 0.1413 | canonical |
| 474 | **POLYSACCHARIDE METABOLIC PROCESS** | 0.5489 | 0 | 0 | GO |
| 66 | **CARBOHYDRATE CATABOLIC PROCESS** | 0.5382 | 0 | 0 | GO |
| 414 | **NEGATIVE REGULATION OF TRANSFERASE ACTIVITY** | 0.5184 | 0 | 0 | GO |
| 311 | **LIPID TRANSPORT** | 0.4952 | 0.011 | 0.4363 | GO |
| 709 | **RESPONSE TO HORMONE STIMULUS** | 0.4714 | 0 | 0 | GO |
| 325 | **AXON GUIDANCE (HSA04360)** | 0.4708 | 0.002 | 0.212 | canonical |
| 796 | **TGF BETA SIGNALING PATHWAY** | 0.3345 | 0.015 | 0.4958 | GO |
| 89 | **CELL MIGRATION** | 0.3224 | 0 | 0 | GO |
| 307 | **LIPID BIOSYNTHETIC PROCESS** | 0.2726 | 0 | 0 | GO |
| 327 | **FOCAL ADHESION (HSA04510)** | 0.2697 | 0.001 | 0.1413 | canonical |
| 342 | **HNF3ALPHA** | 0.2537 | 0 | 0 | TF |
| 398 | **FOXO4** | 0.2275 | 0.01 | 0.4064 | TF |
| **negative scores:** | | | | | |
| 304 | **PROTEASOME (HSA03050)** | -1.4566 | 0.035 | 0.4947 | canonical |
| 68 | **CD40 PATHWAY** | -1.34 | 0 | 0 | canonical |
| 731 | **RIBOSOME BIOGENESIS AND ASSEMBLY** | -1.2146 | 0 | 0 | GO |
| 742 | **RRNA PROCESSING** | -1.1864 | 0 | 0 | GO |
| 481 | **POSITIVE REGULATION OF CELL CYCLE** | -1.1464 | 0 | 0 | GO |
| 461 | **NUCLEOTIDE METABOLISM** | -1.1156 | 0.006 | 0.1696 | canonical |
| 141 | **G2 PATHWAY** | -1.1117 | 0 | 0 | canonical |
| 102 | **DNA REPLICATION REACTOME** | -1.1062 | 0.011 | 0.2915 | canonical |
| 741 | **RRNA METABOLIC PROCESS** | -1.0891 | 0.022 | 0.3193 | GO |
| 74 | **CELL CYCLE (KEGG)** | -1.0795 | 0 | 0 | canonical |
| 464 | **ONE CARBON POOL BY FOLATE** | -1.0775 | 0 | 0 | canonical |
| 647 | **REGULATION OF MITOSIS** | -0.9976 | 0 | 0 | GO |
| 648 | **REGULATION OF MITOTIC CELL CYCLE** | -0.9931 | 0 | 0 | GO |
| 183 | **DNA REPLICATION INITIATION** | -0.966 | 0.043 | 0.4264 | GO |
