# Supplementary material for: A Comprehensive Panel of Three-Dimensional Models for Studies of Prostate Cancer Growth, Invasion and Drug Responses
Source: PLoS One. 2010 May 3;5(5):e10431. doi: 10.1371/journal.pone.0010431 (PMC2862707; doi:10.1371/journal.pone.0010431)
Supplement: Table S6 — Ingenuity Pathway Analysis (IPA) for genes differentially expressed between 2D monolayer and 3D spheroid culture in Matrigel (general effects, 10 cell lines), and B) IPA for differentially expressed genes in PC3 cells, comparing round (day 4+8) with stellate morphology (day 13+15). (0.06 MB DOC) [file pone.0010431.s011.doc]

**Table S6: A) Ingenuity Pathway Analysis (IPA) of 2D monolayer vs. 3D spheroid culture in matrigel (general 3D effects, all 10 cell lines)**

| Top Functions | Score | Focus Molecules | Molecules in Network |
| --- | --- | --- | --- |
| Dermatological Diseases and Conditions, Immunological Disease, Inflammatory Disease | 44 | 27 | AMH, AP1G1, AP1G2, Arginase, BIRC3, CSF2RA, DENR, DYNC1H1, FAM46A, GTF3C1, Ifn gamma, Igm, IL18, IL12 (complex), IL18BP, MHC Class II, MST1R, NCOR2, NFkB (complex), PDCD4, PDXK, PLXNA1, PLXNB1, PNPT1, PPM1B, RIPK4, SLIT2, SQSTM1, SUPT5H, TBC1D17, TFPI, TNFSF14, TNFSF15, Transferase |
| Lipid Metabolism, Small Molecule Biochemistry, Connective Tissue Development and Function | 43 | 27 | ABCC3, ACSS2, ALP, CEBPD, Creb, CYP51A1, DHCR7, DHCR24, EIF2C2, FABP4, FADS1, FADS2, HDL, HMG CoA synthase, HMGCS1, IgG, JUND, LDL, LDLR, LEP, LSS, MVD, NRP1, Oxidoreductase, P38 MAPK, PCSK9, PTGR2, RECQL4, SC5DL, SEMA3E, SQLE, SREBF1, TAPBP, TM7SF2, TNRC6B |
| Cellular Function and Maintenance, Connective Tissue Development and Function, Cellular Development | 38 | 25 | ACACA, BAT2, BMP1, C14ORF153, Calcineurin protein(s), CASP2, CHRNA5, CHSY1, CIAO1, Cytochrome c, FAM177A1, FAM83H, hCG, Histone h4, Hsp90, ITGAV, LRDD, MAGT1, MAN2C1, MIR124, MIR1, MVK, MXD4, MYST3, NGFRAP1, PFKL, Pkc(s), RERE, TDP1, TLN1, UBAP2L, USP49, Vegf, XPNPEP3 |
| Cell Cycle, Embryonic Development, Lipid Metabolism | 35 | 24 | 26s Proteasome, Akt, Alpha actin, AMPK, ATF4, ATP2A2, CABIN1, CAST, Cbp/p300, CHD4, CLOCK, DPYSL3, EP300, EP400, FDFT1, HCFC1, Hdac1/2, HIPK2, HISTONE, HMG20B, HMGCR, MEF2, MEF2D, MIB2, MTA2, N4BP2, OGT, PER2, SETD1A, T3-TR-RXR, Thyroid hormone receptor, TNRC6A, TOP2B, TRIM13, Ubiquitin |
| Cell-To-Cell Signaling and Interaction, Connective Tissue Development and Function | 30 | 22 | ACTN4, Alpha Actinin, Alpha catenin, CALD1, Calmodulin, Calpain, CaMKII, Ck2, CPD, CREB1, Cyclin E, EIF4G1, F-Actin, IGF2R, LIG1, Mapk, Mlc, MYH9, MYL5, Myosin, PCNT, Pdgf, PKD1, PLEC1, PP2A, PPFIBP1, RAD9A, RAVER1, ROCK2, SMG7, SPN, SPTAN1, SPTBN1, UBR4, UPF2 |
| DNA Replication, Recombination, and Repair, Cell Death, Connective Tissue Disorders | 28 | 22 | Actin, BCR, BIK, Caspase, CD79A, CD79B, CFLAR, DAPP1, DUSP4, DUSP8, ERK, FCAR, Growth hormone, H19, IKK (complex), IL17RD, ILF3, Jnk, LPP (includes EG:4026), MAP3K11, MAPK8IP3, MVP, Nfat (family), PI3K, PRKDC, Proteasome, PTPRF, Rac, Sapk, SFRP1, SRGAP3, TAOK2, TCR, TRRAP, WASF2 |
| Cancer, Gastrointestinal Disease, Tumor Morphology | 24 | 18 | ACLY, Adaptor protein 2, AIRE, Ap1, CCR6, CTSZ , ERK1/2, FLNB, FSH, Gsk3, IFN Beta, IL1, IL10, Insulin, Interferon alpha, LILRB1, LZTR1, MCM7, MCM8, MHC Class I (complex), MUC1, NUP214, PDGF BB, Pka, PKM2, PURB, RAB27A, Ras, Smad, SMAD3, SSTR2, STAT, STAT5a/b, Tgf beta, TSPYL2 |

**B) IPA for differentially expressed genes in PC3 cells, comparing round (day 4 and 8) with stellate morphology (day 13 + 15).**

| Top Functions | Score | Focus Molecules | Molecules in Network |
| --- | --- | --- | --- |
| Connective Tissue Disorders, Genetic Disorder, Cellular Movement | 47 | 27 | BEND5, BHLHE40, C5ORF13, COL18A1, COL1A1, COL3A1, COL7A1, Collagen(s), DDIT4, DDR1, DNTTIP1, F11R, ICOSLG, INPPL1, Integrins, Integrin alpha 3 beta 1, ITPKB, LAMA3, Laminin, LOX, LTBP4, MMP13, NCOA7, NFkB (complex), NFKBIZ, P4HA1, Pdgf, PTHLH, SAA@, SPARC, TFPI, Tgf beta, TNFRSF19, TNFRSF25, TRAF5 |
| Infection Mechanism, Cell-mediated Immune Response, Cellular Development | 43 | 26 | 14-3-3, ABTB1, Akt, AMPK, APLN, ARID3A, ATXN1, C10ORF10, CCNG2, CXCR4, EFEMP2, FAM107A, FCGR2A, FOXO3, G alpha i, HHEX, KIAA1217, KRT15, Ldh, Mir125b, NEBL, NRP1, PFKFB3, Pias, PLSCR4, RALGDS, RHPN1, RUNX3, SH3PXD2A, SLC2A1, TCF4, TFF3, Tubulin, Vegf, ZYX |
| Cancer, Cell-To-Cell Signaling and Interaction, Dermatological Diseases and Conditions | 35 | 22 | ABR, APOBEC3F, APOBEC3G, Calpain, ERK, Fgf, Fibrinogen, GRN, Integrin alpha 2 beta 1, Integrin alpha 4 beta 1, Integrin alpha;, Integrin beta;, ITGA5, ITGA6, ITGA10, ITGB2, ITGB4, KISS1R, LAMB2, LAMC1, LPCAT1, Mapk, NISCH, PLA2, PLA2G4C, Pld, Rac, Rap1, RCAN1, SPRY2, SPRY4, TFF2, TNK2, TUBGCP2, UCN |
| Cardiovascular Disease, Genetic Disorder, Respiratory Disease | 32 | 21 | ACCN2, CLK2, ENaC, FCGBP, HISTONE, Hsp70, IFN Beta, IgG, Ikb, IL1, IL1F7, Interferon alpha, IRF9, KLK4, KLK11, Mmp, MMP10, MMP23B, MYB, MZF1, NT5E, P38 MAPK, PI3K, SAT1, SCNN1B, SCNN1G, SERPINE2, SLC12A2, STAT, STAT2, TNFSF10, TRIB2, Trypsin, Ubiquitin, XK |
| Cellular Development, Cellular Growth and Proliferation, | 29 | 20 | ABCB6, ABL1, Actin, ADCY, APBB3, APP, C20ORF46, CAMK2B, CaMKII, CDH1, CELSR3, Ck2, CSPG5, E2F, ENO3, F Actin, HIST2H4A, Histone h3, Histone h4, Hsp90, IL12 (complex), KIFC2, MICAL1, PALM, PAM, PARP10, peptidase, PLD1, Proteasome, Ras, RNA polymerase II, SGSM2, TFF1, TPP1 |
| Cardiovascular System Development and Function, Lymphoid Tissue Structure and Development, | 21 | 15 | ANXA3, APOD, B4GALT3, beta estradiol, BRD2, BZRAP1, cholesterol, CNP, COL4A5, EPHA1, GUCY1A3, GUCY1B3, Hat, HIST2H2AA3, HIST2H2BE, HRAS, ITGAE, LCN2, LHFPL2, MET, MYST4, NELL2, ODZ3, PLXNB3, RAB24, RPS28, SC4MOL, SCNN1A, Soluble guanylate cyclase, SPARC, SPOCK2, SPRY1, SPRY2, TSPO, ZHX2 |
| Infection Mechanism, Organismal Injury and Abnormalities, | 21 | 15 | B4GALNT4, BST2, CASP7, Caspase 3/7, COL9A2, CSGALNACT1, D-galactosamine, ECE1, FAM117B, GNAO1, HOXB6, IFI6, IFI35, IFIH1, IFIT1, IFIT3, IFITM1, IFNA2, IFNB1, IRF9, ISGF3, KIAA0907, MAGED1, MIRN330, OAS1, P2RY5, PLEKHB1, PRNP, retinoic acid, RGS11, RGS7BP, SLC8A3, SPARC, TBXAS1, ULK1 |
