# Supplementary material for: A Comprehensive Panel of Three-Dimensional Models for Studies of Prostate Cancer Growth, Invasion and Drug Responses
Source: PLoS One. 2010 May 3;5(5):e10431. doi: 10.1371/journal.pone.0010431 (PMC2862707; doi:10.1371/journal.pone.0010431)
Supplement: Table S7 — Summary of small molecule inhibitors and drug treatments used in this study, directed against canonical pathways identified by functional gene expression analyses. Abbreviations: IB = invasion block; IAM = impaired acinar morphogenesis; GR = growth reduction; GA = growth arrest; CD = cell death. (0.16 MB DOC) [file pone.0010431.s012.doc]

**Table S7: Compounds and compound treatments.**

| **pathway** | **Compound** | **Target** | **IC50** | **[conc.]** | **effect in 3D (PC3)** | **% inhibition monolayer (PC3)** | **3D effect EP156T** |
| --- | --- | --- | --- | --- | --- | --- | --- |
| **AKT** | (-) Deguelin | AKT, PI3K, ODC | 11.0, 6.9 nM | 10 nM | IB, GA 48h, CD 96h | 4% (100 nM) |  |
|  |  |  |  | 40 nM | IB, GA 48h, CD 72h | 23% (at 400 nM) |  |
|  | 5-(2-Benzothiazolyl)-3-ethyl-2-[2-methylphenylamino)ethenyl]-1-phenyl-1H-benzimidazolium iodide | AKT, FOXO1a |  | 1 µM | severe GR 24h,CD 96h | 62% |  |
|  |  |  |  | 4 µM | severe GR 24h,CD 48h |  |  |
|  | API-2 | AKT (selective) | 5 µM | 0.5 µM | no effects |  | no effects |
|  |  |  |  | 2 µM | partial IB |  | no effects |
|  |  |  |  | 5 µM | partial IB, GR | 26% | GR, IAM |
|  |  |  |  | 20 µM | GR, CD 48h | 61% | severe GR |
|  | API-59CJ-Ome hydrate | AKT (highly selective) | 200 nM | 0.25 µM | GR 96h, IAM |  |  |
|  |  |  |  | 1 µM | GR 72h, IAM |  |  |
|  |  |  |  | 2.5 µM | IB 24h, GR 48h, CD 96h |  |  |
|  |  |  |  | 10 µM | IB 24h, GR 48h, CD 72h |  |  |
|  | FPA 124 | AKT | 2.5 µM | 5 µM | no effects |  |  |
|  |  |  |  | 20 µM | partial IB, GR 48h, |  |  |
|  | Akt1/2 kinase inhibitor | AKT (AKT1, AKT2, AKT3) | 58, 210, and 2120 nM | 60 nM | no effects |  |  |
|  |  |  |  | 240 nM | some GR |  |  |
|  | 10-DEBC | AKT, mTOR,  p70 S6K | 2.5 µM | 5 µM | severe GR 72h, CD 96h |  |  |
|  |  |  |  | 20 µM | severe GR 72h, CD 48h |  |  |
| **PI3K** | 740 Y-P | PI3K (activator) | - | 10 µM | no effects |  |  |
|  |  |  |  | 40 µM | no effects |  |  |
|  | Wortmannin | PI3K | 2-4 nM | 10 nM | no effects |  |  |
|  |  |  |  | 40 nM | no effects |  |  |
|  | PI 103 | PI3K (p110α ) | 8nM | 10 nM | GR | 4% |  |
|  |  |  |  | 40 nM | IB, severe GR 48h | 12% |  |
|  | AS604850 | PI3K (PI3Kγ) |  | 5 µM | no effects | 1% |  |
|  |  |  |  | 20 µM | complete IB, GR | 47% | severe GR |
|  | PI 828 | PI3Kβ, α, δ and γ | 0.098, 0.183, 0.227 and 1.967 | 0.1 µM | some GR |  |  |
|  |  |  |  | 0.4 µM | IB, GR |  |  |
|  | LY294002 | PI3Kβ, α, δ, and γ | 0.31, 0.73, 1.06, 6.60 µM | 0.3 µM |  |  |  |
|  |  |  |  | 1.2 µM | IB, GR 96h |  |  |
| **pathway** | **Compound** | **Target** | **IC50** | **[conc.]** | **3D effect (PC3)** | **monolayer PC3 (% inhibition)** | **3D effect EP156T** |
| **PI3K**  **cont.** | LY 303511 | neg. Control for LY294002 | none | 5 µM | no effects |  |  |
|  |  |  |  | 20 µM | no effects |  |  |
|  | Compound 401 | PI3K, mTOR, ATM and ATR | 0.28 and 5.3 μM | 2.5 µM | no effects |  |  |
|  |  |  |  | 10 µM | partial GR, CD 48h |  | severe GR |
|  | NU7026 | PI3K, DNA-PK, ATM | 13, 0.23, >100 nM | 1 µM | some GR | 10% | no effect |
|  |  |  |  | 4 µM | IB, GR 72h | 10% | no effect |
|  | 2-APB | PI3K, ITPR3 | 42 µM | 10 µM | partial IB, GA 72h | 0% |  |
|  |  |  |  | 40 µM | IB, GA 72h | 25% | rapid CD |
| **IGF1R** | IGF-1 recombinant | IGF1R | 10-200 ng/ml | 0.005 µM | incr. invasion |  |  |
|  |  |  |  | 0.02 µM | incr. invasion |  |  |
|  | IGF-2 recombinant | IGF1R | 10-200 ng/ml | 0.005 µM | incr. invasion |  |  |
|  |  |  |  | 0.02 µM | incr. invasion |  |  |
|  | I-OMe-Tyrphostin AG 538 | IGF1R | 5 µM | 5 µM | no effects |  |  |
|  |  |  |  | 20 µM | no effects |  |  |
|  | Picropodophyllotoxin | IGF1R | 50 - 150 nM | 2.5 µM | rapid CD (< 24h) | 65% (0.1 µM) | CD 24h |
|  |  |  |  | 10 µM | rapid CD (< 24h) | 68% (0.2µM) | CD < 24h |
|  | PQ401 | IGF1R | 12 µM | 10 µM | severe GR 48h | 98% | CD 24-48h |
|  |  |  |  | 40 µM | CD 24h | 100% | CD 24h |
| **JAK/**  **STAT** | Cucurbitacin I | JAK2/STAT3 | n.d. | 2.5 µM | CD 24h | 75% | rapid CD |
|  |  |  |  | 10 µM | rapid CD 24h | 88% |  |
|  | ZM 449829 | JAK3, EGFR, JAK1 | 6.8, 5.0, 4.7 µM | 10 nM |  |  |  |
|  |  |  |  | 40 nM | partial IB |  |  |
|  | WHI-P | JAK3, STAT1 | 1.8 µM | 2.5 µM | partial IB |  |  |
|  |  |  |  | 10 µM | IB |  |  |
| **hedge-hog** | AY 9944 dihydrochloride | hedgehog, SMO | 13 nM; sterol synthesis | 0.05 µM | no effects |  |  |
|  |  |  |  | 0.2 µM | no effects |  |  |
|  | JK 184 | hedgehog, GLI1 | 30 nM | 0.05 µM | CD 72h |  |  |
|  |  |  |  | 0.2 µM | CD 72h |  |  |
|  | Cyclopamine | hedgehog, SMO | 5 µM | 3 µM | GA 72h, partial IB | 11% | n.d. |
|  |  |  |  | 12 µM | GA 72h, complete IB | 49% | GR , CD 7d |
|  | SANT-1 | hedgehog, SMO | 1.2 nM | 10 nM | no effcts |  |  |
|  |  |  |  | 40 nM | no effects |  |  |
|  | SHH Sonic hedgehog, rec. | hedgehog, SMO |  | 1µg/ml | no effects |  |  |
